# Short-term neonatal outcomes in women with gestational diabetes treated using metformin versus insulin: a systematic review and meta-analysis of randomized controlled trials

**DOI:** 10.1007/s00592-022-02016-5

**Published:** 2023-01-03

**Authors:** Bo Sheng, Juan Ni, Bin Lv, Guoguo Jiang, Xuemei Lin, Hao Li

**Affiliations:** 1 Department of Anesthesiology, West China Second University Hospital, Sichuan University, Chengdu, 610041 Sichuan China; 2 Department of Gynecology and Obstetrics, West China Second University Hospital, Sichuan University, Chengdu, 610041 Sichuan China; 3 Key Laboratory of Birth Defects and Related Diseases of Women and Children, Ministry of Education, Sichuan University, Chengdu, 610041 Sichuan China; 4 Department of Hospital Infection Management, The Second Hospital of Chengdu City, Chengdu, 610041 Sichuan China

**Keywords:** Gestational diabetes mellitus, Insulin, Metformin, Neonatal outcomes, Randomized controlled trials

## Abstract

**Aims:**

To expand the evidence base for the clinical use of metformin, we conducted a meta-analysis of randomized controlled trials (RCTs) comparing the efficacy and safety of metformin versus insulin with respect to short-term neonatal outcomes.

**Methods:**

A comprehensive search of electronic databases (PubMed, Embase, Cochrane Library, and Web of Science) was performed. Two reviewers extracted the data and calculated pooled estimates by use of a random-effects model. In total, 24 studies involving 4355 participants met the eligibility criteria and were included in the quantitative analyses.

**Results:**

Unlike insulin, metformin lowered neonatal birth weights (mean difference − 122.76 g; 95% confidence interval [CI] − 178.31, − 67.21; *p* < 0.0001), the risk of macrosomia (risk ratio [RR] 0.68; 95% CI 0.54, 0.86; *p* = 0.001), the incidence of neonatal intensive care unit admission (RR 0.73; 95% CI 0.61, 0.88; *p* = 0.0009), and the incidence of neonatal hypoglycemia (RR 0.65; 95% CI 0.52, 0.81; *p* = 0.0001). Subgroup analysis based on the maximum daily oral dose of metformin indicated that metformin-induced neonatal birth weight loss was independent of the oral dose.

**Conclusions:**

Our meta-analysis provides further evidence that metformin is a safe oral antihyperglycemic drug and has some benefits over insulin when used for the treatment of gestational diabetes, without an increased risk of short-term neonatal adverse outcomes. Metformin may be particularly useful in women with gestational diabetes at high risk for neonatal hypoglycemia, women who want to limit maternal and fetal weight gain, and women with an inability to afford or use insulin safely.

**Supplementary Information:**

The online version contains supplementary material available at 10.1007/s00592-022-02016-5.

## Introduction

Gestational diabetes mellitus (GDM) is a common complication during pregnancy and is defined as any glucose intolerance that occurs or is diagnosed for the first time during pregnancy [1]. GDM develops in about 5% to 14% of all pregnancies and is associated with certain pregnancy-related complications and a long-term risk of diabetes in both the mother and offspring [2]. With the establishment of the two-child policy and epidemic of obesity in China, the incidence of GDM has been increasing, resulting in a heavy economic burden on the public health care system and individuals [3]. According to the latest data reported by the International Diabetes Federation in 2021, about one in six live births (20 million) is affected by high plasma glucose concentration during pregnancy, and GDM accounts for 83.6% of these cases of hyperglycemia [4].

Women with uncontrolled GDM have higher-risk pregnancies, and some adverse effects of GDM may also affect the fetus, including fetal anomalies, macrosomia (birth weight of > 4000 g), fetal distress, metabolic disorders, growth imbalance, hyperbilirubinemia, and some long-term complications [5]. Traditionally, insulin has been the gold standard for the treatment of GDM because it cannot cross the placenta and allows for precise glucose control. However, insulin therapy has several disadvantages, including the need for multiple injections, risks of hypoglycemia and hyperbilirubinemia, the rising cost of insulin, and the lack of affordability [6]. These disadvantages suggest that current treatment regimens fall short of optimizing outcomes. Metformin is a commonly used oral antihyperglycemic drug in clinical practice with excellent efficacy in terms of glycemic control and weight loss, good tolerance, and a reasonable price [7]. Several organizations currently support its use as an alternative to insulin [8, 9]. However, recent long-term studies of offspring have provided conflicting results. Two follow-up studies of children aged 2 to 9 years whose mothers had gestational diabetes showed that several growth parameters tended to be larger in metformin-exposed offspring than in offspring exposed to insulin. These growth parameters included weight, body mass index, triceps skinfold, waist and arm circumferences and body fat percent, and they were also associated with cardio-metabolic disease in later life [10, 11]. This has slowed the clinical use of metformin as a substitute for insulin in the treatment of GDM.

We therefore performed this updated meta-analysis to compare the efficacy and safety of metformin versus insulin with respect to short-term neonatal outcomes in the treatment of GDM. The objective of our study was to determine whether metformin is superior to insulin in terms of altering neonatal growth outcomes and inducing neonatal adverse outcomes during treatment of GDM. Addressing this issue is particularly important because the number of pregnancies exposed to metformin is increasing worldwide.

## Methods

This systematic review and meta-analysis are reported in accordance with the Preferred Reporting Items for Systematic Reviews and Meta-Analyses (PRISMA) Statement and was registered at the International Prospective Register of Systematic Reviews (CRD42022330187) [12].

### Search strategy

A systematic literature search of PubMed, Embase, the Cochrane Library, and Web of Science (last search was updated on 1 May 2022) was performed using prespecified terms (Supplemental Text S1) with no filters and no language or location restrictions. We also searched for additional eligible trials in previously published meta-analyses on related topics.

### Inclusion and exclusion criteria

Studies that met the following criteria were included: (1) The population comprised pregnant women with GDM, (2) The interventions were metformin (with or without extra insulin treatment) and insulin, (3) The study included one or more neonatal outcomes, and (4) The study design was a randomized controlled trial (RCT). We excluded studies involving pregnant women with pre-existing diabetes, and duplicate studies published in different journals were included only once.

### Definitions of neonatal outcomes

The neonatal outcomes included neonatal growth outcomes and neonatal adverse outcomes. The neonatal growth outcomes were birth weight, birth height, macrosomia (≥ 4000 g), large for gestational age (LGA) (birth weight at the > 90th percentile), and small for gestational age (SGA) (birth weight at the < 10th percentile). The neonatal adverse outcomes were neonatal hypoglycemia, admission to the neonatal intensive care unit (NICU), hyperbilirubinemia, respiratory distress syndrome, premature birth, congenital anomalies, abnormal pH of the umbilical cord, abnormal Apgar score at 5 min, neonatal death, neonatal sepsis, and birth trauma.

### Data collection and management

The titles, abstracts, citation information, and descriptor terms of the publications identified through the search strategy were screened. Full-text articles of all selected abstracts were obtained, and two reviewers (Bo Sheng and Juan Ni) independently assessed all the full-text articles for eligibility to determine the final study selection. Any disagreements between the two authors were settled by group discussion until a consensus was reached. We designed a data extraction form to collect relevant information including the authors, year of publication, country, number of patients, definition of gestational diabetes, patient characteristics, and interventions.

### Risk of bias and quality assessment

We used the Cochrane Collaboration’s tool to assess the risk of bias in terms of the following seven aspects: (1) Random sequence generation (selection bias), (2) Allocation concealment (selection bias), (3) Blinding of participants and personnel (performance bias), (4) Blinding of outcome assessment (detection bias), (5) Incomplete outcome data (attrition bias), (6) Selective reporting (reporting bias), and (7) Other bias. We classified these aspects as low risk of bias, uncertain risk of bias, or high risk of bias.

We assessed the quality of evidence in these studies by using the GRADE profiler (GRADEpro GDT) [13]. The GRADE system was used to assess the study limitations (risk of bias), inconsistency, indirectness, imprecision, and publication bias across the body of evidence to derive an overall summary of the quality of evidence, which was classified each as high, moderate, low, or very low.

### Statistical analysis

The standardized mean difference (SMD) was calculated using the mean and standard deviation for continuous variables. The risk ratio (RR) was calculated for dichotomous variables with 95% confidence intervals (CIs). The meta-analysis was performed using Review Manager (RevMan) version 5.4.1 (Nordic Cochrane Centre, Copenhagen, 2014), and Egger’s test was used to assess publication bias through the ‘metafor’ package in R version 3.5.1 [14]. The studies were determined to be heterogenous if *I*^2^ > 50% and *p* < 0.1. A sensitivity analysis was performed by excluding each study one by one to evaluate the credibility of the pooled results. A prespecified subgroup analysis was also performed to explore the sources of heterogeneity. Potential publication bias was assessed by the application of contour-enhanced funnel plots and Egger’s linear regression test at the *p* < 0.05 level of significance. If publication bias was indicated, we further evaluated the number of missing studies by trim-and-fill analysis and recalculated the pooled risk estimate with the addition of those missing studies. Except where otherwise specified, a *p* value of < 0.05 was considered statistically significant.

## Results

### Literature search and study characteristics

**Fig. 1:**
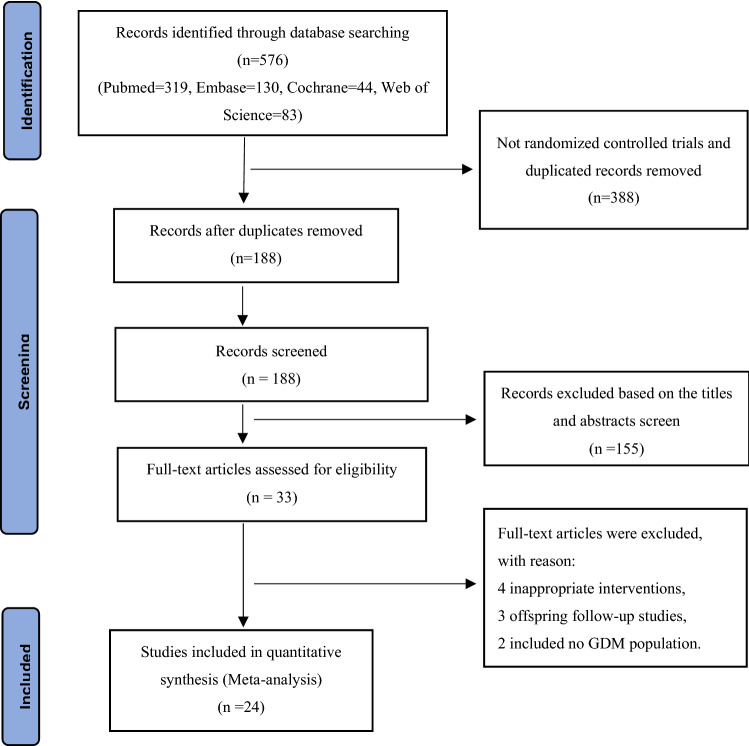
PRISMA flow diagram

In total, 576 studies were retrieved through PubMed, Embase, the Cochrane Library, and Web of Science. After removal of duplicates and title/abstract screening, 188 trials underwent full text assessment, after which the full set of eligibility criteria was applied. After full text evaluation, 24 studies remained eligible for inclusion in this review. The process of study selection is illustrated in Fig. 1. As shown in Table 1, 24 RCTs involving 4355 patients with GDM were included to estimate the impact of metformin versus insulin on neonatal outcomes [15–38]. The earliest study began in 2001, and the latest study was completed in 2021. Five studies each were conducted in Iran [23, 30–32, 35],Egypt [15, 18, 20, 22, 37], and Pakistan [16, 17, 21, 27, 38]; three in Finland [26, 28, 34]; and one each in Australia [19], India [36], Spain [29], Brazil [33], New Zealand [24], and the USA [25]. In this meta-analysis, we mainly focused on the daily oral dose of metformin in pregnant women with GDM. Three studies among the 24 RCTs did not report the dose of metformin [19, 23, 30], and the remaining 21 studies were included for further subgroup analysis.

**Table 1:** Main characteristics of RCTs included in the meta-analysis

Author, year	Country	Study period	Participants	Participants enrolled			Dose		No. of Neonatal outcomes
				Metformin	Escalating to Insulin§	Insulin	Metformin (mg/day)	Insulin (units/kg/day)	
Abdullah, 2021	Egypt	Oct 2019 to Apr 2021	Women aged 21–35 yr; Singleton; Gestational age, 20–28 wks	94	5	100	500–2500	0.7	7
Ainuddin, 2014	Pakistan	Dec 2008 to Dec 2010	Women aged 20–46 yr; Singleton; Gestational age, 20–36 wks	43	32	75	500–2500	0.9	15
Arshad, 2017	Pakistan	2010 to 2012	NR	25	NR	25	1500	08–0.9	5
Ashoush, 2016	Egypt	Jan 2013 to Nov 2014	Gestational age, 26–32 wks	47	11*	48	1000–2500	0.7	8
Barrett, 2013	Australia	NR	Singleton	236	97	242	NR	NR	13
Eid, 2018	Egypt	Mar 2016 to Jun 2017	Women aged 18–42 yr; Singleton; Gestational age, 22–30 wks	113	2	116	500–2500	0.5	17
Ghomian, 2018	Iran	NR	Women aged 18–40 yr; Singleton; Gestational age. 24–28 wks	143	30	143	NR	NR	6
Gamal, 2018	Egypt	Feb 2016 to Jan 2017	NR	58	5*	58	1500–2500	1.0	3
Hassan, 2012	Pakistan	Dec 2008 to Dec 2010	Singleton; Gestational age, 20–35 wks	75	18*	75	500–3000	NR	10
Hamadani, 2017	Pakistan	NR	Singleton	30	NR	30	500–2000	NR	2
Huhtala, 2020	Finland	Jun 2006 to Dec 2010	NR	110	23*	110	500–2000	NR	6
Ijas, 2010	Finland	Jun 2005 to Jun 2009	Singleton; Gestational age, 12–34 wks	32	15*	50	750–2250	NR	12
Jahanshahi, 2020	Iran	2017 to 2018	Singleton; Gestational age, 20–34 wks	30	3	30	NR	NR	2
Picón-César, 2021	Spain	Oct 2016 to June 2019	Women aged 18–45 yr; Singleton; Gestational age, 14–35 wks	70	24*	97	425–2500	0.3	15
Mesdaghinia, 2013	Iran	NR	Women aged 18–45 yr; Singleton; Gestational age, 24–34 wks	100	22	100	500–2500	0.5	13
Moore, 2007	USA	2001 to 2004	Gestational age, 24–30 wks	32	0	31	500–2000	0.7	6
Niromanesh, 2012	Iran	Dec 2010 to Jan 2012	Women aged 18–40 yr; Singleton; Gestational age, 20–34 wks	80	11*	80	500–2500	0.7	14
Rowan, 2008	New Zealand	NR	Women aged 18–45 yr; Singleton; Gestational age, 20–33 wks	363	168*	370	500–2500	NR	15
Ruholamin, 2014	Iran	2011	Women aged 18–45 yr. Singleton; Gestational age,t 24–33 wks	50	2	50	500–1500	0.2	13
Saleh, 2016	Egypt	Nov 2012 to Dec 2014	Gestational age, 26–34 wks	67	NR	70	500–3000	0.7–1	12
Somani, 2016	India	Feb 2014 to Jul 2015	Women aged 18–35 yr. Singleton; Gestational age, 24–34 wks	32	1	33	500–2000	NR	11
Spaulonci, 2013	Brazil	Nov 2007 to Jan 2010	Singleton	47	12*	47	1700–2250	0.4	11
Tertti, 2013	Finland	Jun 2006 to Dec 2010	Singleton; Gestational age, 22–34 wks	110	23*	110	500–2000	NR	12
Wasim, 2019	Pakistan	Feb 2016 to Dec 2017	Singleton; Gestational age, 22–34 wks	137	34*	141	1000–2500	0.7–0.8	11

*NR* No Reported

^§^ indicates glycemic control is not achieved by maximum metformin dose, and insulin is added

* represents the participants are included in the metformin group for pooled analysis

Supplemental Fig. S1 provides a summary of the risk of bias for each included study. No selection bias, attrition bias, or selective bias was present in any of the RCTs, indicating relatively high quality. Because insulin was given by injection and metformin was given orally, all the included studies involved open allocation, which did not affect the short-term neonatal outcomes because these were all objective. The quality of the evidence (GRADE) for the neonatal outcomes of interest, including neonatal birth weight, macrosomia, LGA, SGA, birth height, NICU admission, and neonatal hypoglycemia, was very low to moderate. The GRADE system evidence for the above outcomes and reasons for upgrade and downgrade are shown in Table 2.

**Table 2:** Grading of Recommendations, Assessment, Development, and Evaluations (GRADE) summary of neonatal outcomes of meta-analysis

Metformin vs. insulin for gestational diabetes mellitus Patient population: patients with gestational diabetes mellitus Intervention: metformin Comparison: insulin						
Outcomes	Anticipated absolute effects		RR/SMD (95%CI)	No. of Participants (Studies)	Certainty of the evidence (GRADE)	Comments
	Risk with Insulin	Risk with Metformin				
Birthweight			SMD -0.33 (-0.5 to -0.17)	4174 (22 RCTs)	⨁⨁◯◯ Low	Most of researches have limitations in methodology Unexplained heterogeneity
Macrosomia	137 per 1,000	93 per 1,000	RR 0.68 (0.54 to 0.86)	3484 (20 RCTs)	⨁⨁⨁◯ Moderate	Researches have limitations in methodology
LGA	188 per 1,000	162 per 1,000	RR 0.86 (0.73 to 1.02)	2843 (12 RCTs)	⨁⨁⨁◯ Moderate	Most of researches have limitations in methodology
SGA	75 per 1,000	75 per 1,000	RR 1.00 (0.77 to 1.30)	2812 (12 RCTs)	⨁⨁◯◯ Low	Most of researches have limitations in methodology Unexplained heterogeneity
Birth Height			SMD -0.09 (-0.27 to -0.08)	1084 (3 RCTs)	⨁◯◯◯ Very low	Researches have limitations in methodology Very few RCTs lead to imprecision of estimate Unexplained heterogeneity
NICU admission	207 per 1000	151 per 1000	RR 0.73 (0.61 to 0.88)	3527 (18 RCTs)	⨁⨁⨁◯ Moderate	Most of researches have limitations in methodology
Hypoglycemia	164 per 1000	107 per 1000	RR 0.65 (0.54 to 0.84)	3670 (20 RCTs)	⨁⨁◯◯ Low	Most of researches have limitations in methodology 2. There is a possibility of publication bias in these studies

GRADE Working Group grades of evidence

*High quality*: Further research is very unlikely to change our confidence in the estimate of the effect

*Moderate quality*: Further research is likely to have an important impact on our confidence in the estimate of the effect and may change the estimate

*Low quality*: Further research is very likely to have an important impact on our confidence in the estimate of the effect and is likely to change the estimate

*Very low quality*: We are very uncertain about the estimate

*RCTs* Randomized controlled trials; *CI* confidence interval; *RR* risk ratio; *SMD* Std mean difference

### Neonatal birth weight and macrosomia

**Fig. 2:**
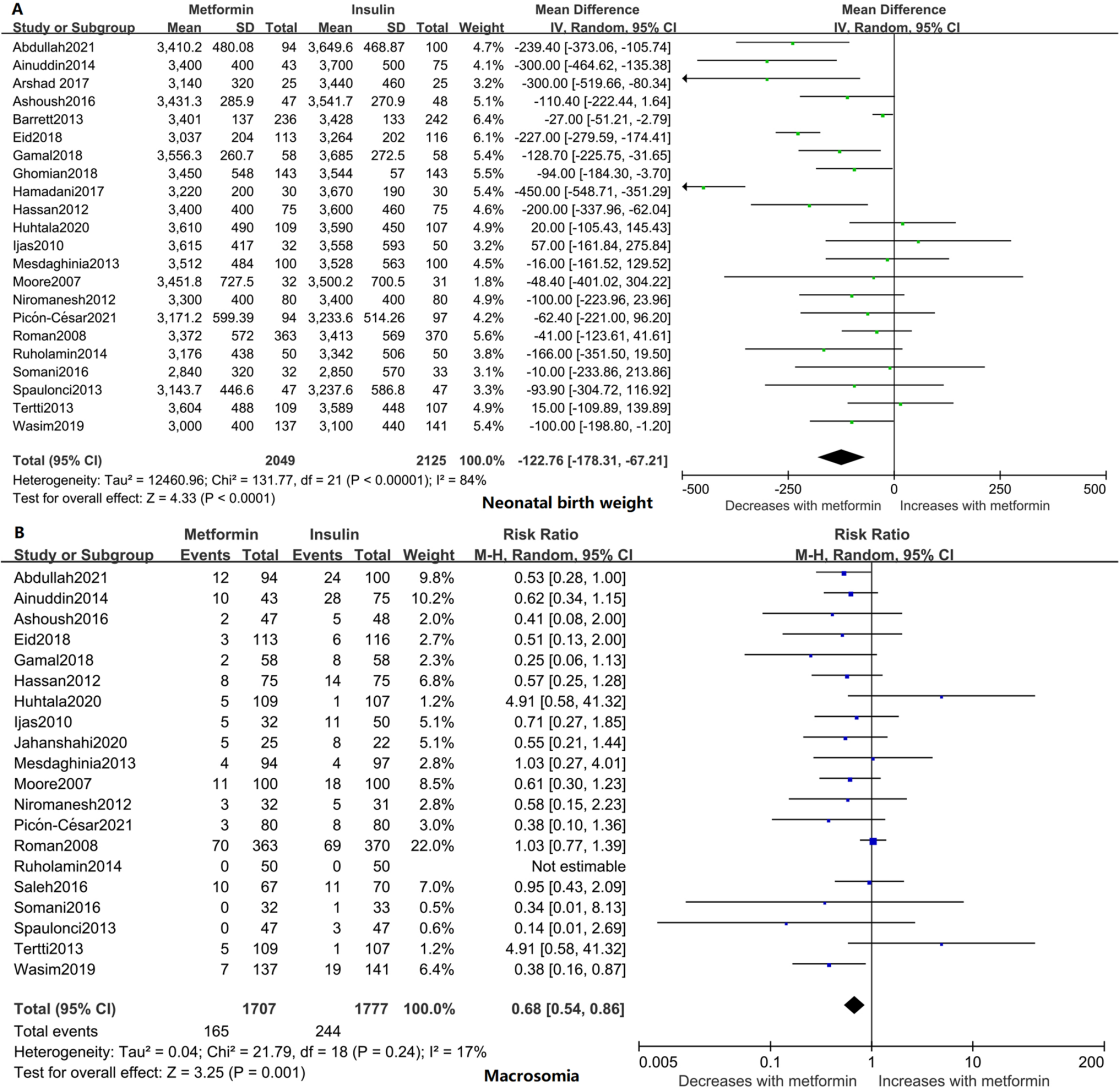
Forest plots for neonatal growth outcomes. **A** Neonatal birth weight. **B** Macrosomia

Twenty-two studies involving 4174 neonates reported the neonatal birth weight. [15–19, 21–23, 25–38] The results indicated that the birth weights of neonates whose mothers were treated with metformin were significantly lower than those of neonates whose mothers were treated with insulin during pregnancy (95% CI − 178.31, − 67.21; *I*^2^ = 84%; *p* < 0.0001) (Fig. 2A). On average, metformin-exposed neonates weighed 122.76 g less than those whose mothers received insulin. Similar to the birth weight in the metformin-exposed group, metformin also lowered the risk of macrosomia by 30% compared with the insulin-exposed group based on 20 studies (RR 0.75; 95% CI 0.54, 0.86; *I*^2^ = 17%; *p* = 0.001) (Fig. 2B) [15, 17, 18, 20, 22, 24–38].

**Fig. 3:**
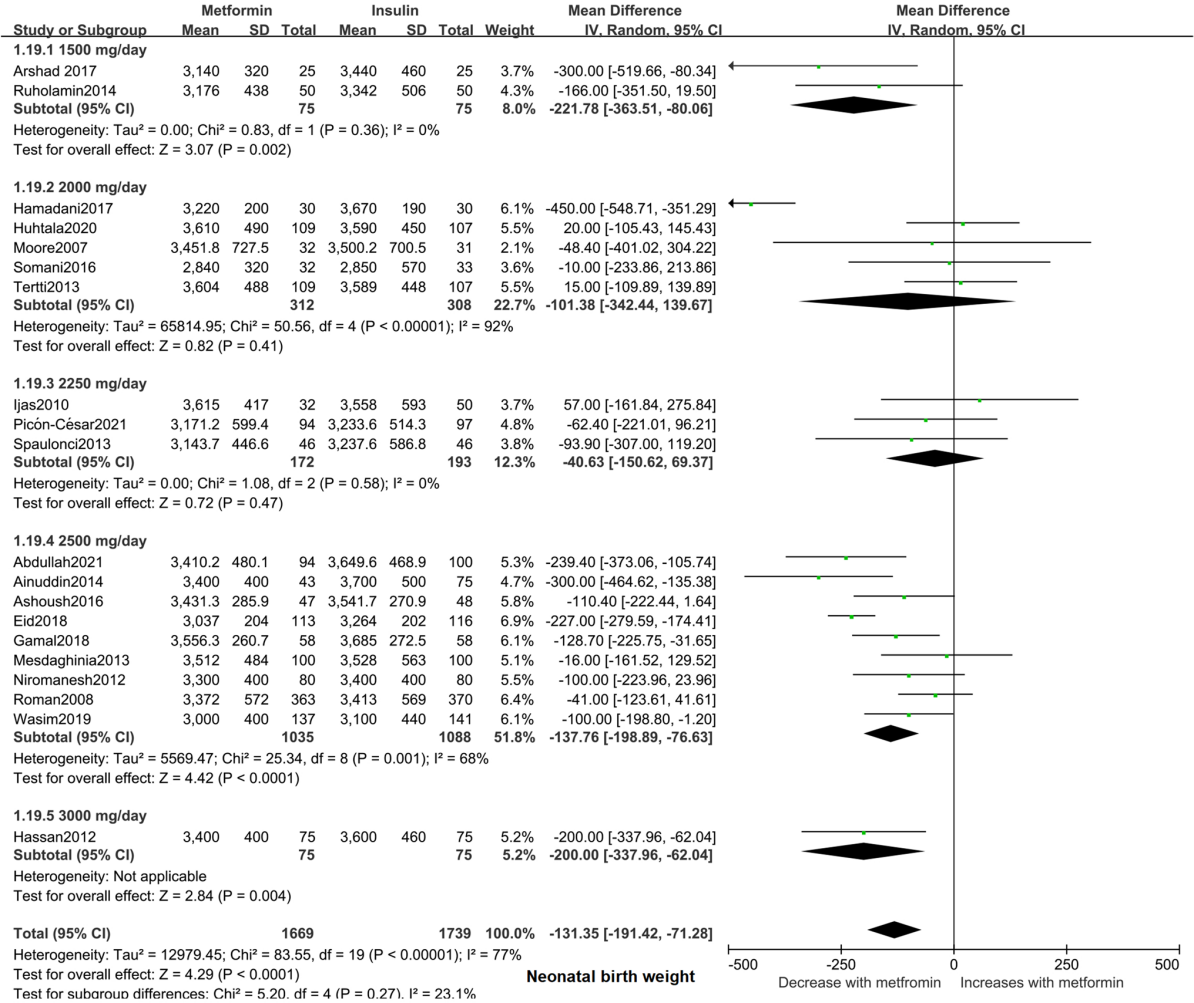
Forest plot for subgroup analysis of neonatal birth weight. Data are expressed as mean difference (random-effects model) and 95% CI

To explore potential source of heterogeneity among the studies, we carried out several sensitivity analyses (Supplemental Fig. S2). Nevertheless, significant heterogeneity (*I*^2^ = 74%) was still present among the studies after we excluded one study from the analysis [21]. Next, 20 studies involving 3408 neonates were included in a subgroup analysis of birth weight [15–18, 21, 22, 24–35, 37, 38], and we found that the neonates whose mothers were treated with a maximum oral dosage of metformin of 1500 mg/day (95% CI − 363.51, − 80.06; *I*^2^ = 0%; *p* = 0.002), 2500 mg/day (95% CI − 198.89, − 76.63; *I*^2^ = 68%; *p* < 0.001), and 3000 mg/day (95% CI − 337.96, − 62.04; *p* = 0.004) had obviously lower birth weights than those of neonates whose mothers were treated with insulin. However, the birth weight of neonates born to mothers treated with a maximum oral dosage of metformin of 2000 mg/day (95% CI − 342.44, 139.67; *I*^2^ = 92%; *p* = 0.41) and 2250 mg/day (95% CI − 150.62, 69.37; *I*^2^ = 0%; *p* = 0.47) showed no significant difference between the groups (Fig. 3).

To assess the potential publication bias of neonatal birth weight, we used the ‘metafor’ package of R software for Egger’s test. Our results showed that the funnel plot of neonatal birth weight was asymmetrical (Supplemental Fig. S3A), and Egger’s test indicated possible publication bias (*p* = 0.008) (Supplemental Table S1). Next, we used trim-and-fill analysis to recalculate our pooled risk estimate; the results suggested no publication bias (*p* = 0.28), and the funnel plot also became symmetrical (Supplemental Fig. S3B).

### Other neonatal growth outcomes

**Fig. 4:**
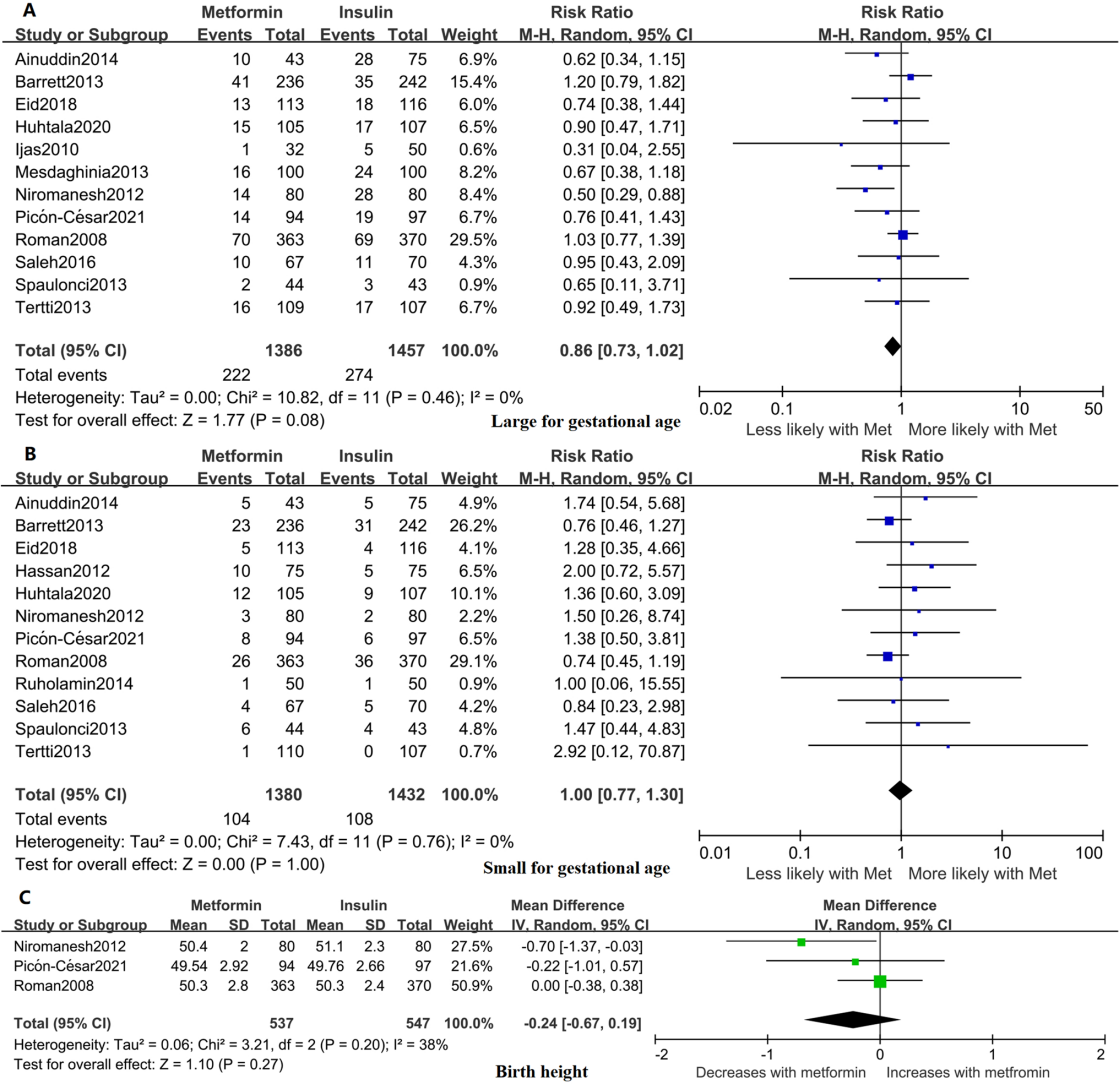
Forest plots for other neonatal growth outcomes. **A** Large for gestational age (LGA). **B** Small for gestational age (SGA). **C** Neonatal birth height

Twelve studies reported the frequency of LGA and SGA [17, 19, 20, 22, 24, 26, 28, 29, 31–34], and three studies reported the neonatal height [24, 29, 31]. The results suggested no difference in the risk of being born LGA (RR 0.86; 95% CI 0.73, 1.02; *I*^2^ = 0%; *p* = 0.08), the risk of being born SGA (RR 1.00; 95% CI 0.77, 1.30; *I*^2^ = 0%; *p* = 1.0), or the neonatal height (95% CI − 0.67, 0.19; *I*^2^ = 38%; *p* = 0.27) between metformin and insulin exposure (Fig. 4). No evidence of publication bias was observed by Egger’s test in other neonatal growth outcomes (Supplemental Fig. S3 and Table S1).

### Neonatal adverse outcomes

**Fig. 5:**
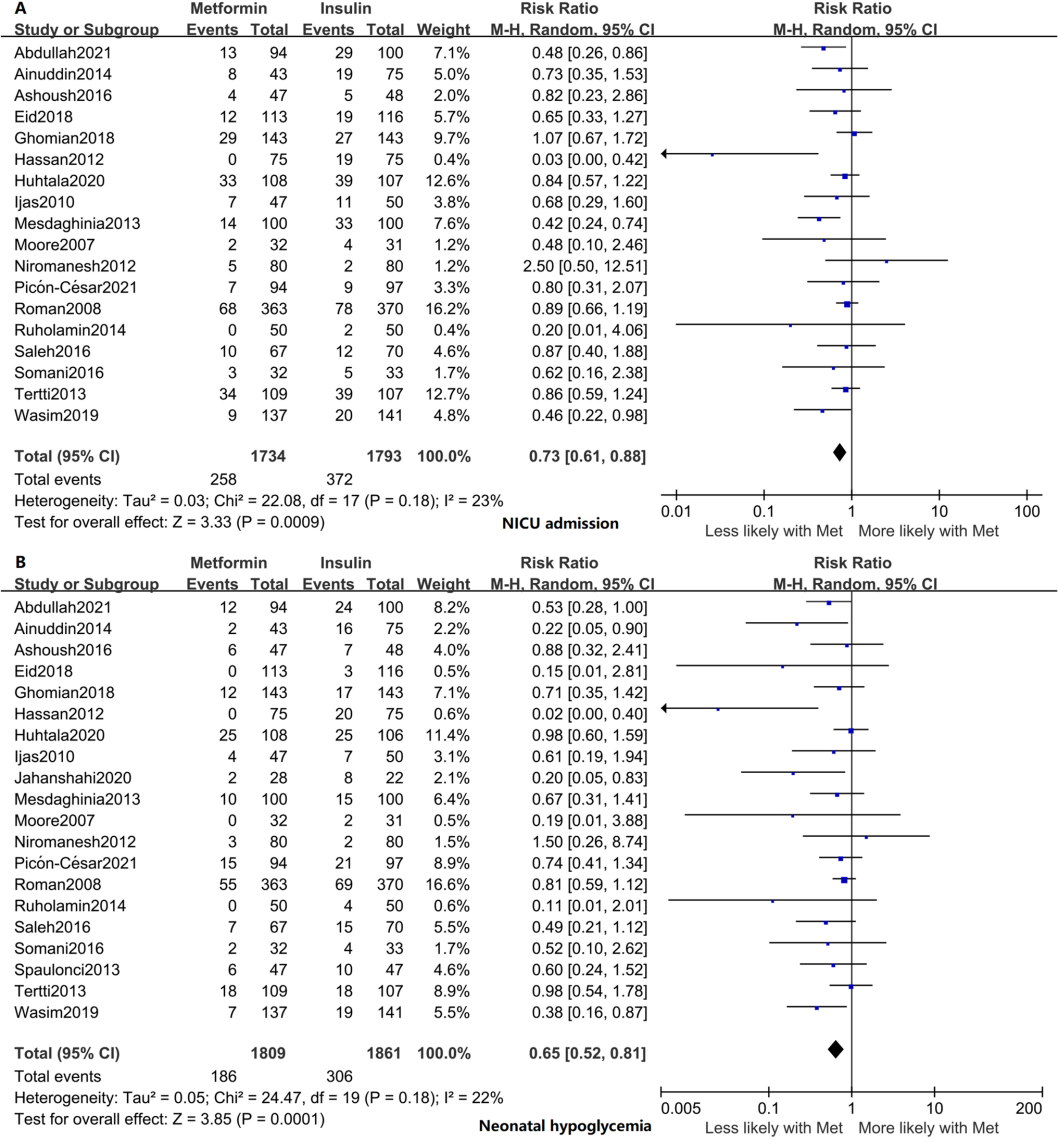
Forest plots for neonatal adverse outcomes. **A** NICU admission. **B** Neonatal hypoglycemia

Eighteen studies involving 3527 neonates reported the incidence of NICU admission [15, 17, 19, 20, 22–29, 31, 32, 34–36, 38], and the results indicated a lower incidence in metformin-exposed than insulin-exposed neonates (RR 0.73; 95% CI, 0.61, 0.88; *I*^2^ = 23%; *p* = 0.0009) (Fig. 5A). Moreover, 20 studies involving 3670 neonates were included in the analysis of neonatal hypoglycemia [15, 17, 18, 20, 22–36, 38]. The results showed that insulin-exposed neonates had a higher incidence of hypoglycemia than metformin-exposed neonates (RR 0.65; 95% CI 0.52, 0.81; *I*^2^ = 22%; *p* = 0.0001) and that metformin lowered the risk of neonatal hypoglycemia by 45% compared with the insulin-exposed group (Fig. 5B). We used contour-enhanced funnel plots and Egger’s linear regression test to assess the potential publication bias of NICU admission and neonatal hypoglycemia (Supplemental Fig. S3D and S3F). Egger’s test indicated no publication bias for NICU admission, but neonatal hypoglycemia was associated with possible publication bias (*p* = 0.006) (Supplemental Table S1). We used trim-and-fill analysis to recalculate our pooled risk estimate of neonatal hypoglycemia, which suggested no publication bias (*p* = 0.71) (Supplemental Fig. S3E).

There were no significant differences in the other neonatal adverse outcomes, including respiratory distress syndrome (14 studies) (RR 0.71; 95% CI, 0.51, 0.99; *I*^2^ = 0%; *p* = 0.07), an abnormal Apgar score at 5 min (15 studies) (RR 0.00; 95% CI − 0.15, 0.16; *I*^2^ = 59%; *p* = 0.95), hyperbilirubinemia (9 studies) (RR 0.88; 95% CI 0.69, 1.12; *I*^2^ = 0%; *p* = 0.29), congenital anomalies (9 studies) (RR 0.73; 95% CI 0.44, 1.22; *I*^2^ = 0%; *p* = 0.23), preterm birth (11 studies) (RR 1.08; 95% CI 0.78, 1.50; *I*^2^ = 22%; *p* = 0.63), an abnormal pH of the umbilical cord (5 studies) (RR 0.01; 95% CI − 0.00, 0.01; *I*^2^ = 0%; *p* = 0.14), neonatal death (10 studies) (RR 0.52; 95% CI 0.13, 2.18; *I*^2^ = 0%; *p* = 0.37), neonatal sepsis (4 studies) (RR 0.71; 95% CI 0.34, 1.45; *I*^2^ = 0%; *p* = 0.34), and birth trauma (6 studies) (RR 0.92; 95% CI 0.57, 1.49; *I*^2^ = 0%; *p* = 0.74) (Supplemental Figs. S4 and S5).

## Discussion

In this systematic review and meta-analysis, we found that neonates exposed to metformin in utero weighed less at birth than those whose mothers were exposed to insulin. The risk of macrosomia is substantially lower (by 30%) when GDM is treated with metformin than with insulin, and there is no concomitant increase in the risk of being born SGA or LGA. Despite being born at lower average birth weights, neonates of metformin-treated women do not have an increased incidence of neonatal adverse outcomes. In contrast, metformin significantly lowers the risk of neonatal hypoglycemia and the incidence of NICU admission.

It is well accepted that the fetuses of obese women with GDM have a higher risk of developing macrosomia than those of women with GDM of normal weight [39]. Some recent meta-analyses showed that weight gain during pregnancy was significantly lower in women with GDM who received metformin than in those who received insulin [4, 7]. Whether metformin-induced weight loss in women with GDM leads to a significant reduction in the incidence of fetal macrosomia remains unclear. Our results provide evidence that metformin can also effectively control neonatal birth weight and reduce the incidence of fetal macrosomia. In particular, there is growing evidence that macrosomia is likely to be associated with shoulder dystocia, brachial plexus injury, delayed motor development, and a higher risk of obesity or diabetes later in life [7, 40]. Moderate neonatal birth weight control may effectively reduce and avoid some complications related to macrosomia, especially for pregnant women with GDM. To explore the relationship between neonatal birth weight and the oral dose of metformin, we performed a subgroup analysis of neonatal birth weight based on the maximum daily oral dose of metformin. We found that a maximum oral dosage of metformin of 1500, 2500, and 3000 mg/day was associated with neonatal birth weight loss, but there was no significant difference in an oral dosage of metformin of 2000 and 2250 mg/day. These results suggest that metformin-induced neonatal birth weight loss occurs independently of the oral dose of metformin. This is consistent with the previous finding that a low dosage of metformin (< 1000 mg/day), but not a high dosage, had significant efficacy for body mass index control or weight loss in adolescents [41].

Macrosomic fetuses in women with diabetes develop a unique pattern of overgrowth involving central deposition of subcutaneous fat in the abdominal and interscapular areas with skeletal growth remaining largely unaffected [40, 42]. During early gestation, the embryo expresses very low levels of organic cation transporters, making metformin likely to be safe in the first trimester. However, metformin can easily cross the placenta via organic cation transporters in the second and third trimesters and may reach near-maternal concentrations in the fetus [43]. In addition to lowering blood glucose concentration, metformin has a variety of intracellular effects including inhibition of mitochondrial respiration and effects on the nutrient-sensing pathway by both adenosine monophosphate-activated protein kinase and mammalian target of rapamycin mechanisms [44–47]. Moreover, in the Metformin in Women with Type 2 Diabetes in Pregnancy (MiTy) trial, the lower neonatal adiposity in the metformin group led to a lower incidence of fetal macrosomia [48]. Therefore, the significant metformin-induced reduction in the incidence of macrosomia may be related to the inhibition of fetal fatty acid synthesis. This effect of metformin differentiates its dose-dependent hypoglycemic effect, the underlying mechanism of which remains to be explored.

In accordance with previous meta-analyses [7, 51, 52], the incidence of NICU admission and hypoglycemia were also significantly reduced in our study. The rates of NICU admission are mainly influenced by fetal physiologic compromise, including preterm birth, hypoglycemia, respiratory distress syndrome, and neonatal jaundice. In our meta-analysis, the infants born to mothers treated with insulin needed additional management for hypoglycemia, which is partly associated with an increase in NICU admission. Neonatal hypoglycemia is one of the most common metabolic disorders of the newborn and is due to hyperinsulinemia of the fetus in response to maternal hyperglycemia in utero [49]. Fetal hypoglycemia can also lead to more serious complications such as seizures and serious brain injury [50]. Notably, metformin significantly lowered the risk of neonatal hypoglycemia by 44% in our meta-analysis, and it may reduce the risk of neonatal brain injury. The use of metformin may not harm the fetus during pregnancy and may be safer in the neonatal period with potentially beneficial effects.

A major strength of our meta-analysis is our provision of a complete overview of the effect of maternal metformin exposure on neonatal growth outcomes and neonatal adverse outcomes. We included 24 studies, which is a higher number than included in previous analyses; additionally, all of these studies were RCTs, which greatly reduced the likelihood of recall and selection biases. Moreover, a subgroup analysis by the different daily doses of metformin for treatment of GDM and an investigation of the relationship between the maternal oral dose of metformin and neonatal birth weight were carried out for the first time. Furthermore, we assessed potential publication bias by contour-enhanced funnel plots and Egger’s test, the results of which suggested that our results regarding neonatal outcomes were not affected by publication bias. This increases the confidence in our findings.

Our study has several limitations that merit further discussion. First, the possibility of confounding factors in several studies cannot be completely ruled out. For example, women who had poor glycemic control with metformin and required extra insulin therapy were included in the metformin-treated group in some studies, which might cause selection bias. However, the proportion of metformin-treated women requiring insulin supplementation ranged from 8.6% to 46.8% (average, 16.2%) of the total metformin-treated women. Moreover, these patients used a lower total insulin dose than those treated with insulin alone. Therefore, we believe that such selection bias may not have influenced the overall outcomes of the studies. Second, data on neonatal growth outcomes and neonatal adverse outcomes were unavailable or incompletely reported in most of the included studies, restricting us from performing a more detailed relevant analysis and obtaining more comprehensive results. Finally, although subgroup and sensitivity analyses were performed to explore the potential sources of heterogeneity in neonatal birth weight, the cause of the high heterogeneity remains unclear.

In conclusion, the results of this meta-analysis add to the evidence that metformin may be particularly useful in women with GDM at high risk for neonatal hypoglycemia, women who want to limit maternal and fetal weight gain, or women with an inability to afford or use insulin safely. Metformin can effectively lower neonatal birth weight and the incidences of macrosomia, neonatal hypoglycemia, and NICU admission compared with insulin without an increased risk of neonatal adverse outcomes. Whether the effect of metformin on neonatal birth weight is associated with the oral dose of metformin requires further investigation in large-scale trials.

## Supplementary Information

Below is the link to the electronic supplementary material.

Text S1. Database search strategies. (A) PubMed. (B) Ovid Embase. (C) Web of Science. (D) Cochrane Library. (DOCX 21 kb)

Table S1. The Egger’s test of neonatal outcomes in the meta-analysis. (DOCX 11 kb)

Fig. S1. Summary of risk of bias for each included study. +, low risk of bias; ?, unclear risk; -, high risk. (TIF 4812 kb)

Fig. S2. Leave-one-out sensitivity analysis of neonatal birth weight. Data are expressed as mean difference (random-effects model) and 95% CI. (PDF 936 kb)

Fig. S3. Funnel plots to assess publication bias of neonatal outcomes. (A) Neonatal birth weight. (B) Neonatal birth weight after trim-and-fill analysis. (C) Macrosomia. (D) Neonatal hypoglycemia. (E) Neonatal hypoglycemia after trim-and-fill analysis. (F) NICU admission. (G) LGA. (H) SGA. (I) Neonatal birth height. (PDF 873 kb)

Fig. S4. Forest plots for neonatal adverse outcomes. (A) Respiratory distress syndrome. (B) Hyperbilirubinemia. (C) Abnormal pH of umbilical cord. (F) Preterm birth. (PDF 2197 kb)

Fig. S5. Forest plots for neonatal adverse outcomes. (A) Apgar score at 5 minutes. (B) Congenital anomalies. (C) Neonatal death. (D) Neonatal sepsis. (E) Birth trauma. (PDF 2421 kb)

## Data Availability

Data will be available upon request of the corresponding author.
