# Supplementary material for: Short-term neonatal outcomes in women with gestational diabetes treated using metformin versus insulin: a systematic review and meta-analysis of randomized controlled trials
Source: Acta Diabetol. 2023 Jan 3;60(5):595–608. doi: 10.1007/s00592-022-02016-5 (PMC10063481; doi:10.1007/s00592-022-02016-5)
Supplement: Supplementary file 1 — Text S1. Database search strategies. (A) PubMed. (B) Ovid Embase. (C) Web of Science. (D) Cochrane Library. (DOCX 21 kb) [file 592_2022_2016_MOESM1_ESM.docx]

**A) Search criteria for PubMed:**

((((((((((Dimethylbiguanidine[Title/Abstract]) OR (Dimethylguanylguanidine[Title/Abstract])) OR (Glucophage[Title/Abstract])) OR (Metformin Hydrochloride[Title/Abstract])) OR (Hydrochloride, Metformin[Title/Abstract])) OR (Metformin HCl[Title/Abstract])) OR (HCl, Metformin[Title/Abstract])) OR ("Metformin"[Mesh])) OR (((((((((((("Insulin"[Mesh]) OR (Insulin, Regular[Title/Abstract])) OR (Regular Insulin[Title/Abstract])) OR (Soluble Insulin[Title/Abstract])) OR (Insulin, Soluble[Title/Abstract])) OR (Insulin A Chain[Title/Abstract])) OR (Sodium Insulin[Title/Abstract])) OR (Insulin, Sodium[Title/Abstract])) OR (Novolin[Title/Abstract])) OR (Iletin[Title/Abstract])) OR (Insulin B Chain[Title/Abstract])) OR (Chain, Insulin B[Title/Abstract]))) AND (((((((Diabetes, Gestational[Title/Abstract]) OR (Diabetes, Pregnancy-Induced[Title/Abstract])) OR (Diabetes, Pregnancy Induced[Title/Abstract])) OR (Pregnancy-Induced Diabetes[Title/Abstract])) OR (Gestational Diabetes[Title/Abstract])) OR (Diabetes Mellitus, Gestational[Title/Abstract])) OR (Gestational Diabetes Mellitus[Title/Abstract]))) AND (randomized controlled trial[Publication Type] OR randomized[Title/Abstract] OR placebo[Title/Abstract])

**B) Search criteria for Ovid Embase:**

(1) metformin.mp.

(2) metformin.ti,ab.

(3) exp *metformin/

(4) (2) or (3)

(5) (gestation* adj3 diabet*).ti,ab.

(6) exp *pregnancy diabetes mellitus/

(7) (pregnan* adj3 diabet*).ti,ab.

(8) (5) or (6) or (7)

(9) (4) and (8)

(10) ((maternal* or gestation* or pregnan*) adj3 diabet*).ti,ab.

(11) (6) or (10)

(12) (4) or (11)

C**) Search terms for Web of Science:**

Metformin AND Insulin AND Gestational Diabetes Mellitus

D**) Search terms for the Cochrane Database:**

Metformin AND Insulin AND Gestational Diabetes Mellitus
