# Supplementary material for: Short-term neonatal outcomes in women with gestational diabetes treated using metformin versus insulin: a systematic review and meta-analysis of randomized controlled trials
Source: Acta Diabetol. 2023 Jan 3;60(5):595–608. doi: 10.1007/s00592-022-02016-5 (PMC10063481; doi:10.1007/s00592-022-02016-5)
Supplement: Supplementary file 2 — Table S1. The Egger’s test of neonatal outcomes in the meta-analysis. (DOCX 11 kb) [file 592_2022_2016_MOESM2_ESM.docx]

**Table S1. The Egger’s test of neonatal outcomes in this meta-analysis**

| Outcomes | Egger’s test (*p* value) | After trim-and-fill § (*p* value) | Publication bias |
| --- | --- | --- | --- |
| Birth weight | 0.008 | 0.28 | (-) |
| Macrosomia | 0.27 |  | (-) |
| LGA | 0.06 |  | (-) |
| SGA | 0.06 |  | (-) |
| Birth height | 0.19 |  | (-) |
| NICU admission | 0.06 |  | (-) |
| Hypoglycemia | 0.006 | 0.71 | (-) |

§ The application of trim-and-fill analysis recalculates the *p* value.
