# Supplementary figures and images for: Short-term neonatal outcomes in women with gestational diabetes treated using metformin versus insulin: a systematic review and meta-analysis of randomized controlled trials
Source: Acta Diabetol. 2023 Jan 3;60(5):595–608. doi: 10.1007/s00592-022-02016-5 (PMC10063481; doi:10.1007/s00592-022-02016-5)

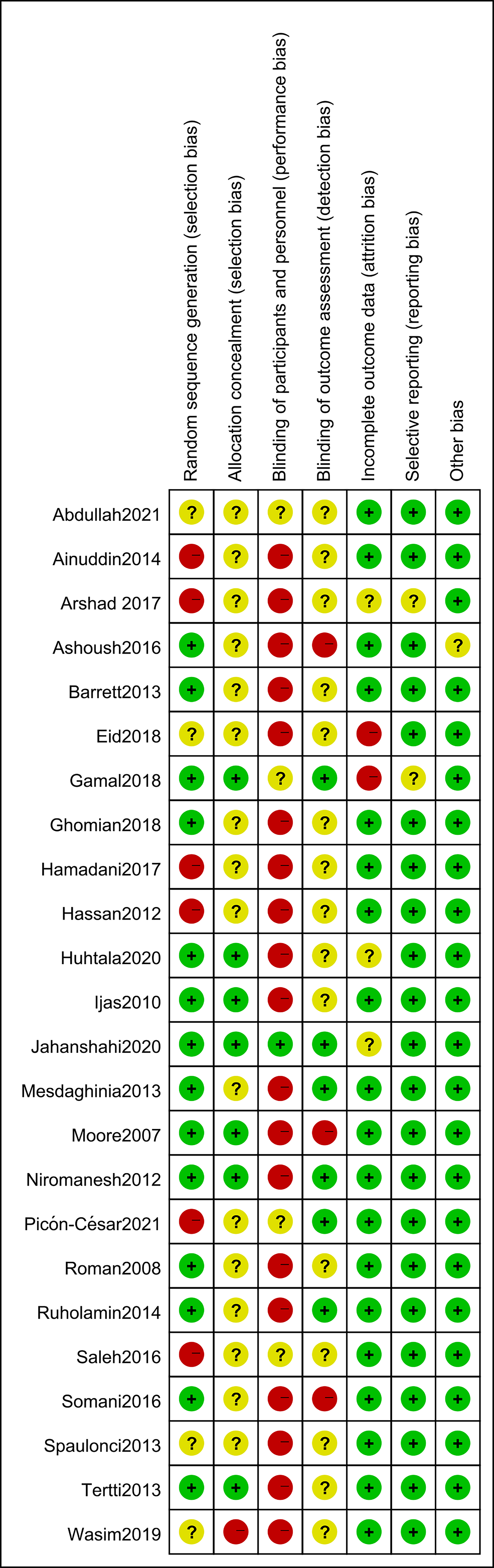

Supplement: Supplementary file 3 — Fig. S1. Summary of risk of bias for each included study. +, low risk of bias; ?, unclear risk; -, high risk. (TIF 4812 kb) [file 592_2022_2016_MOESM3_ESM.tif]

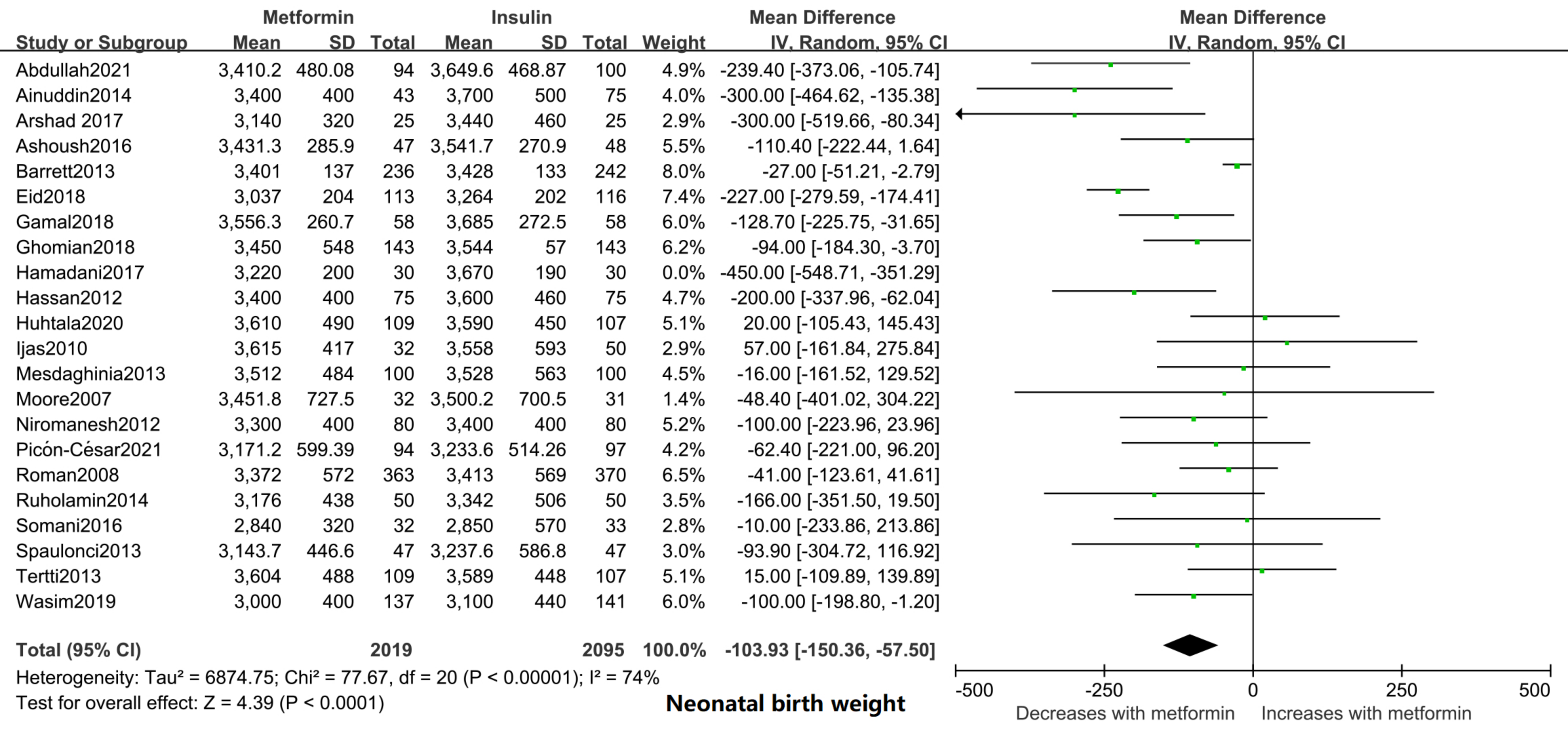

Supplement: Supplementary file 4 — Fig. S2. Leave-one-out sensitivity analysis of neonatal birth weight. Data are expressed as mean difference (random-effects model) and 95% CI. (PDF 936 kb) [file 592_2022_2016_MOESM4_ESM.pdf]

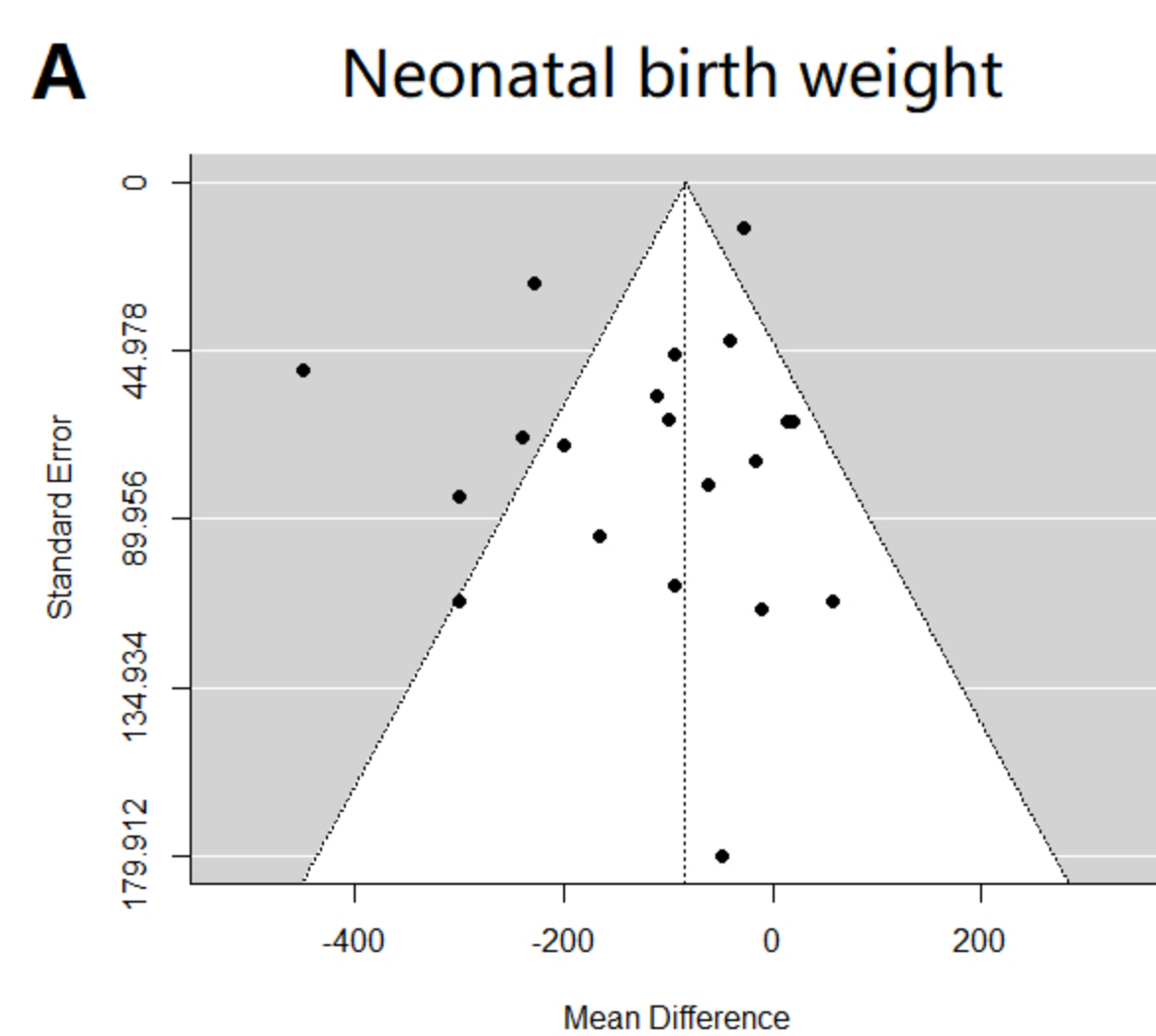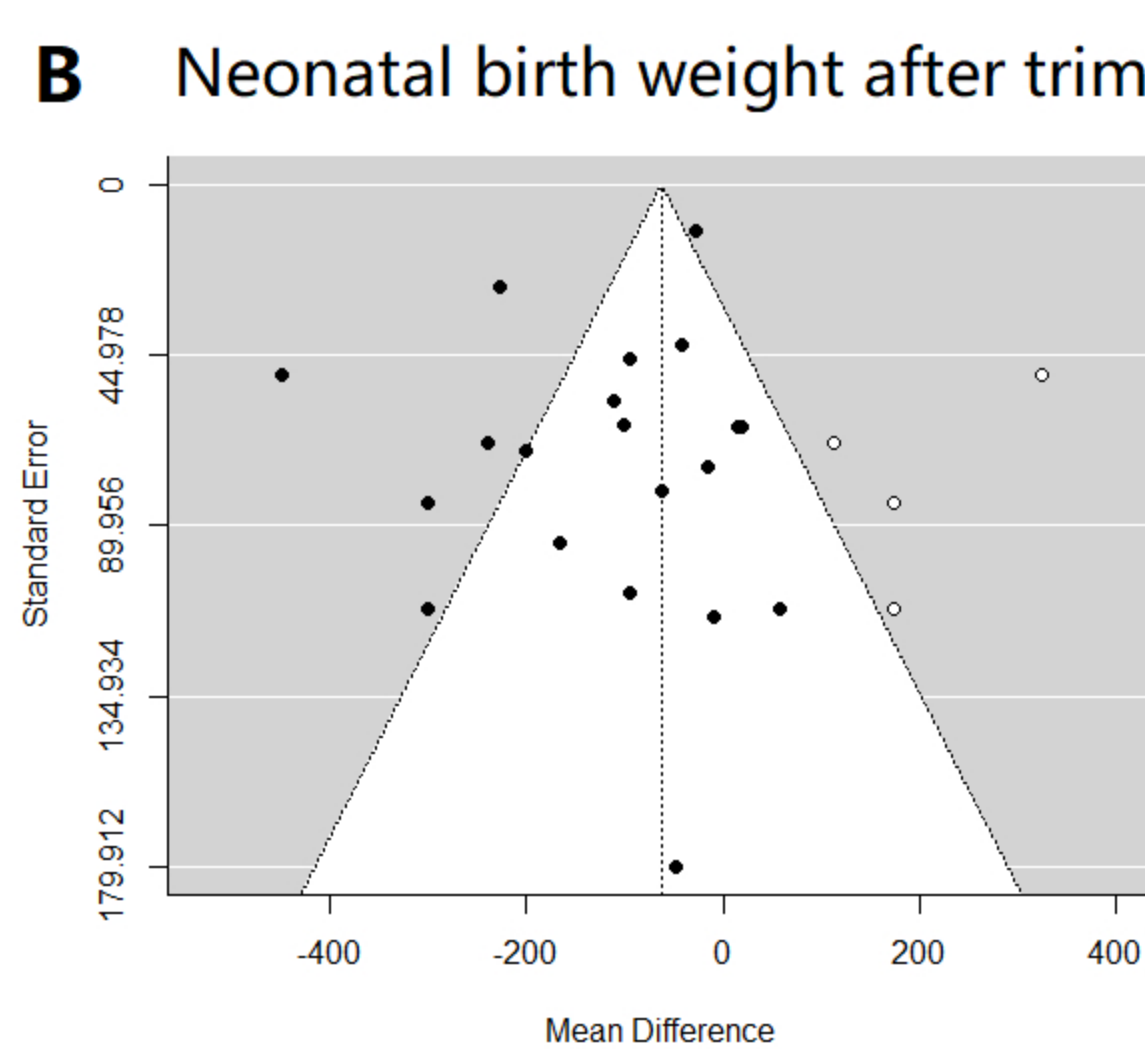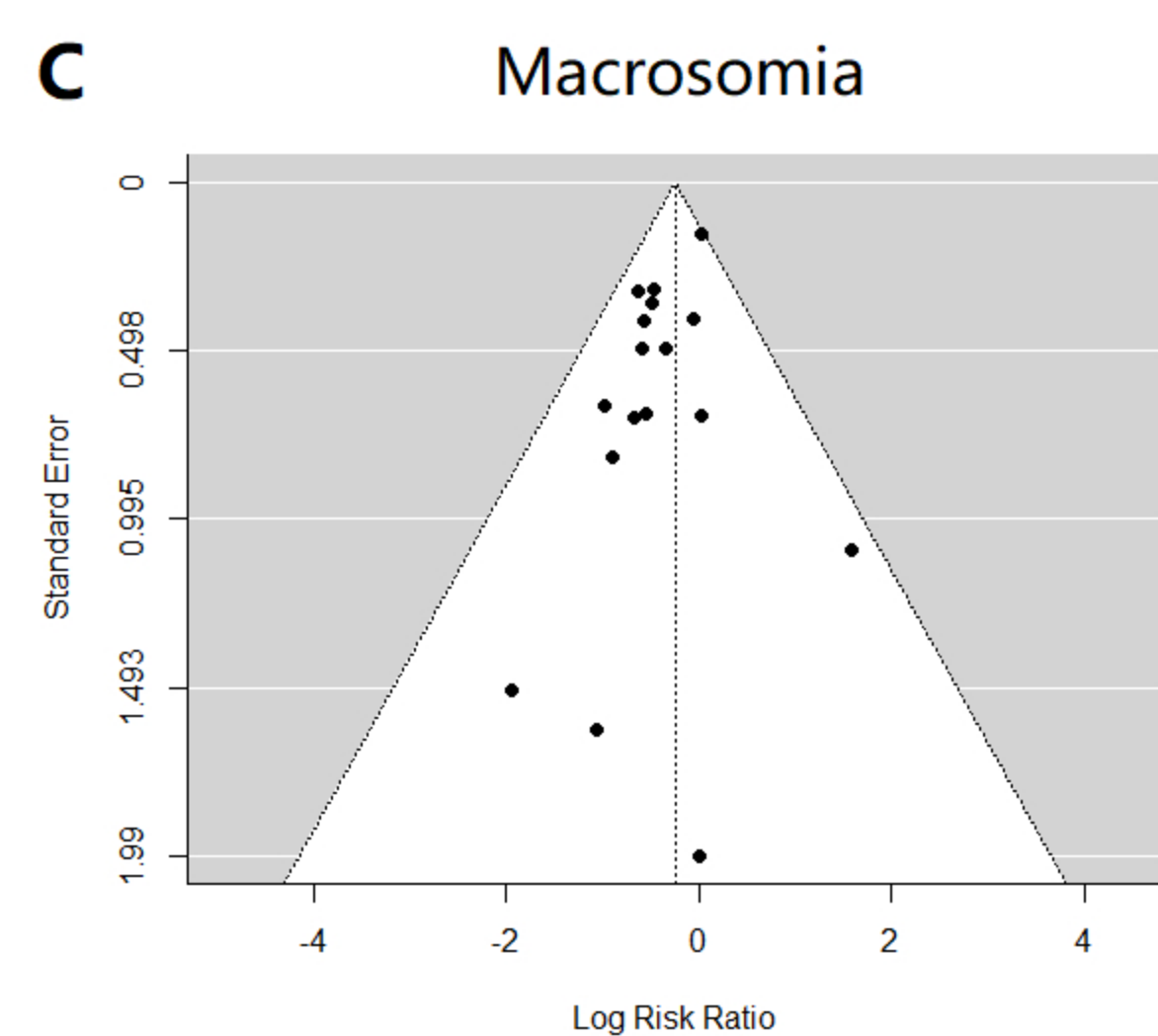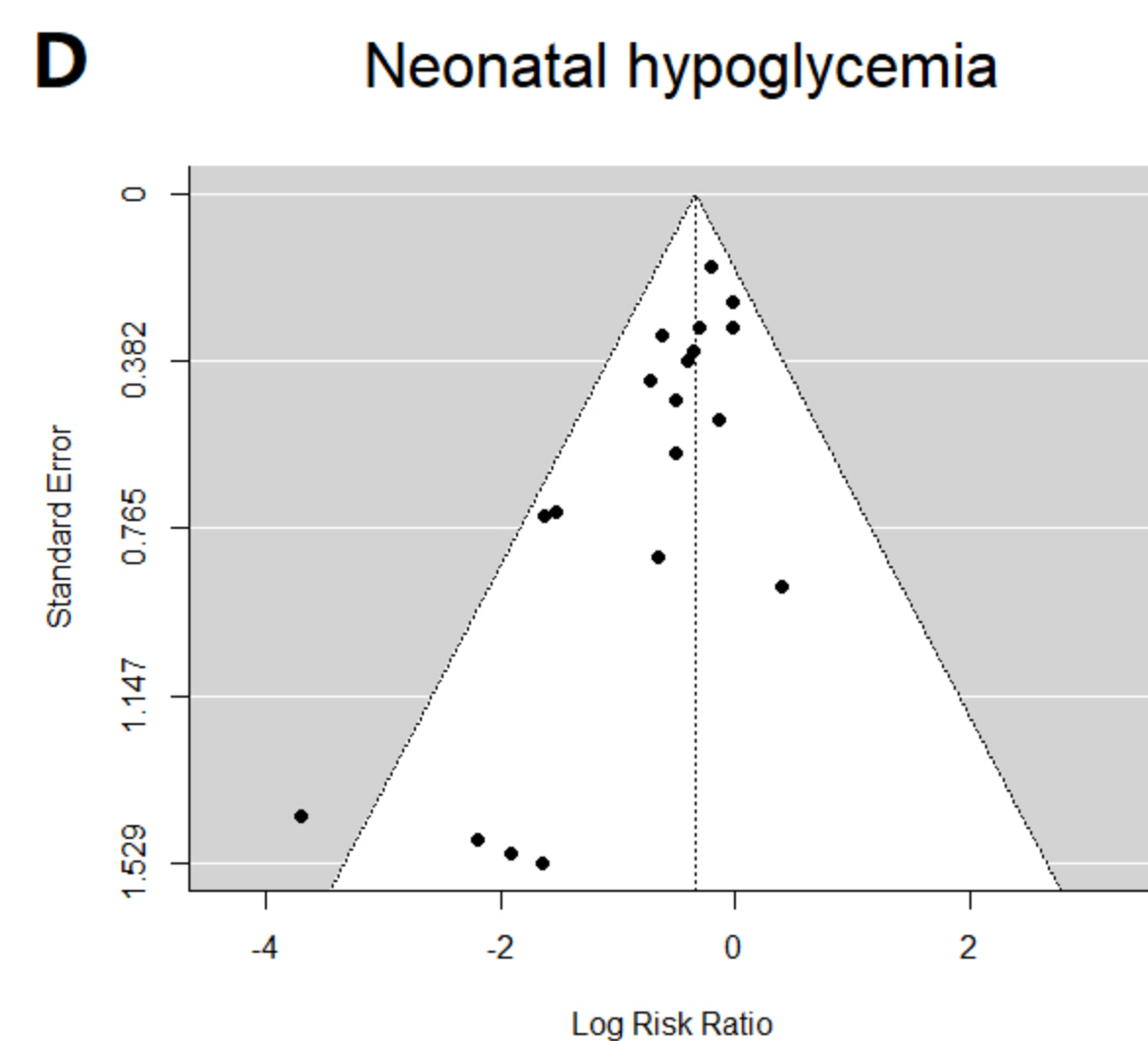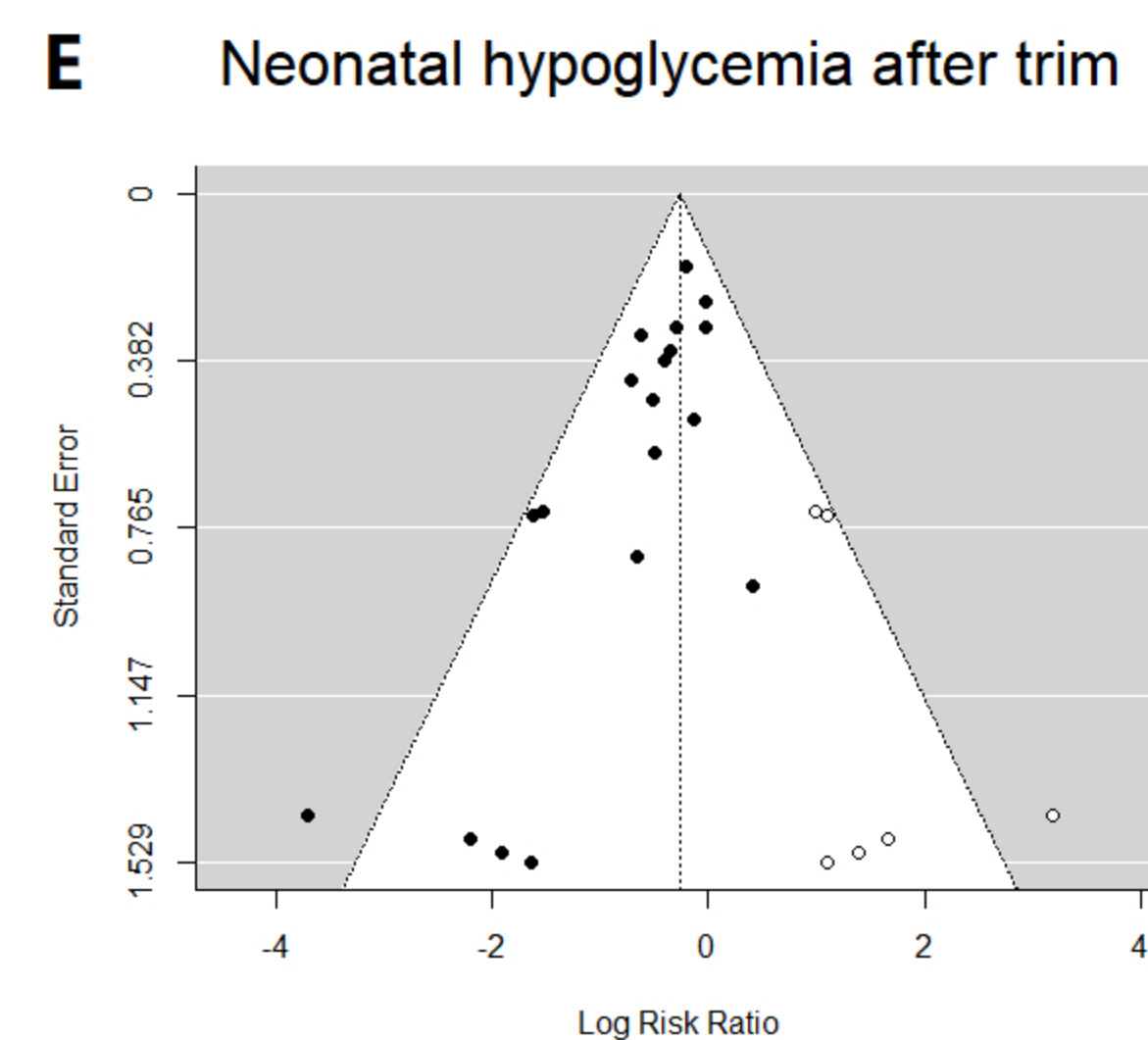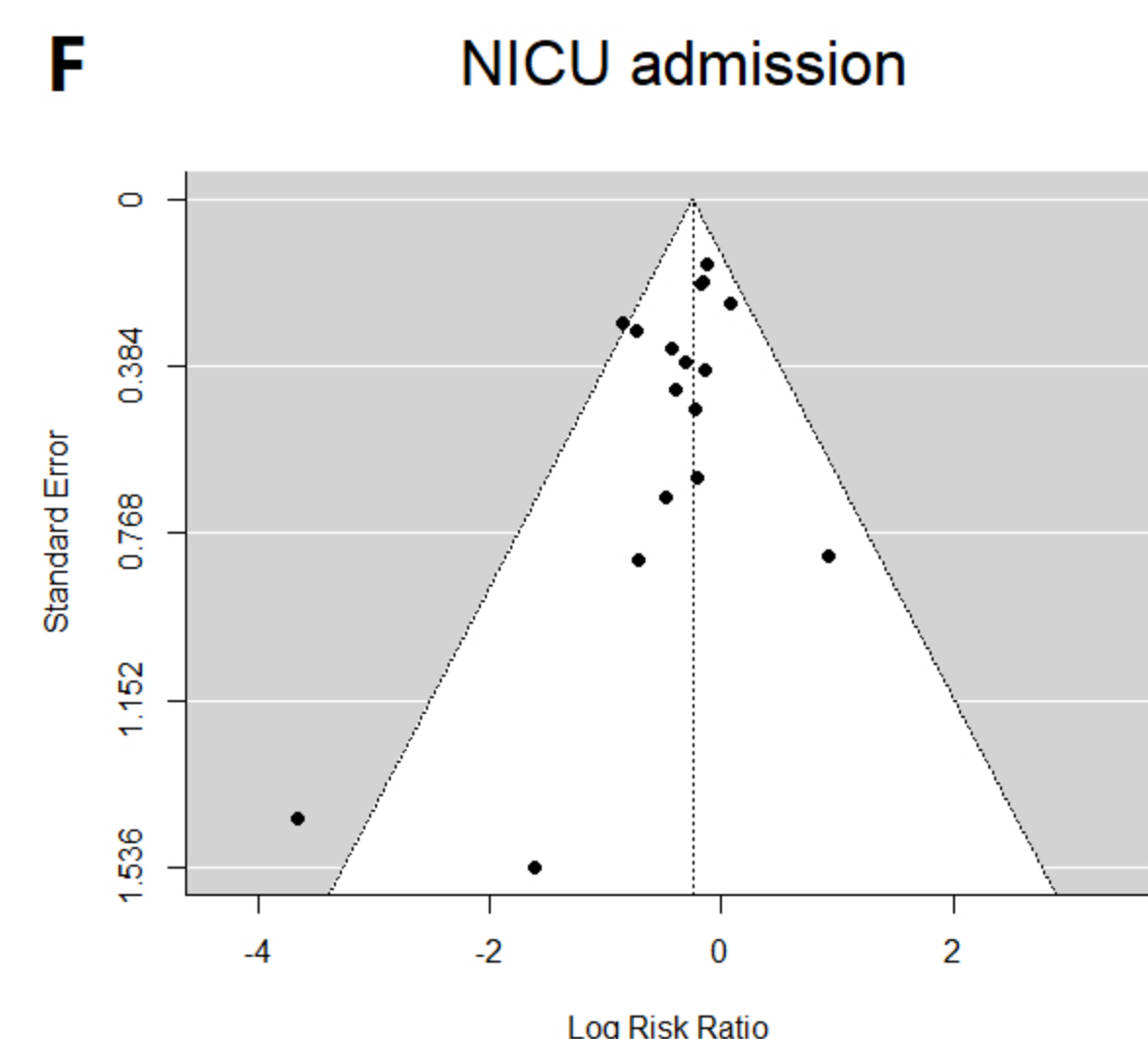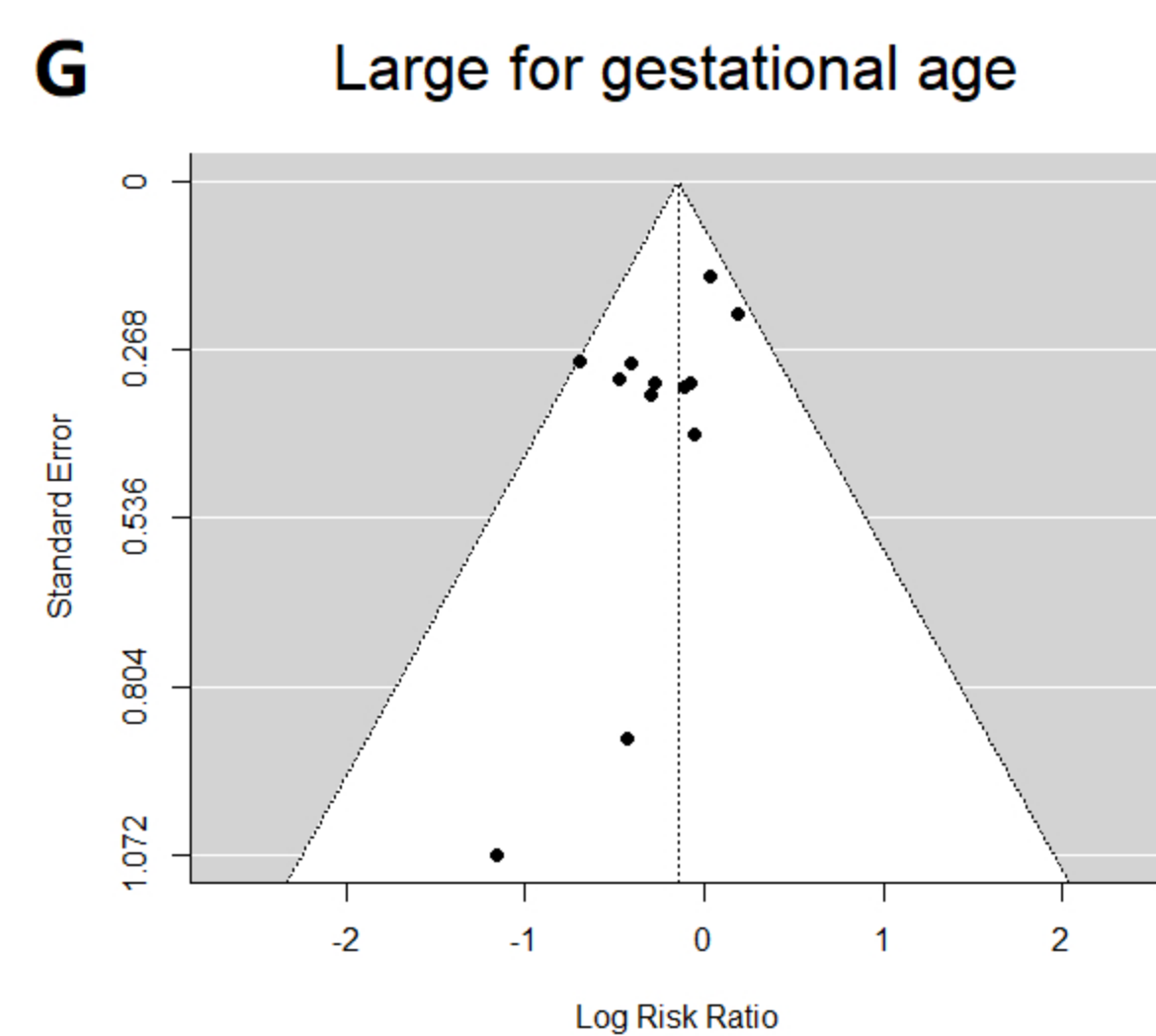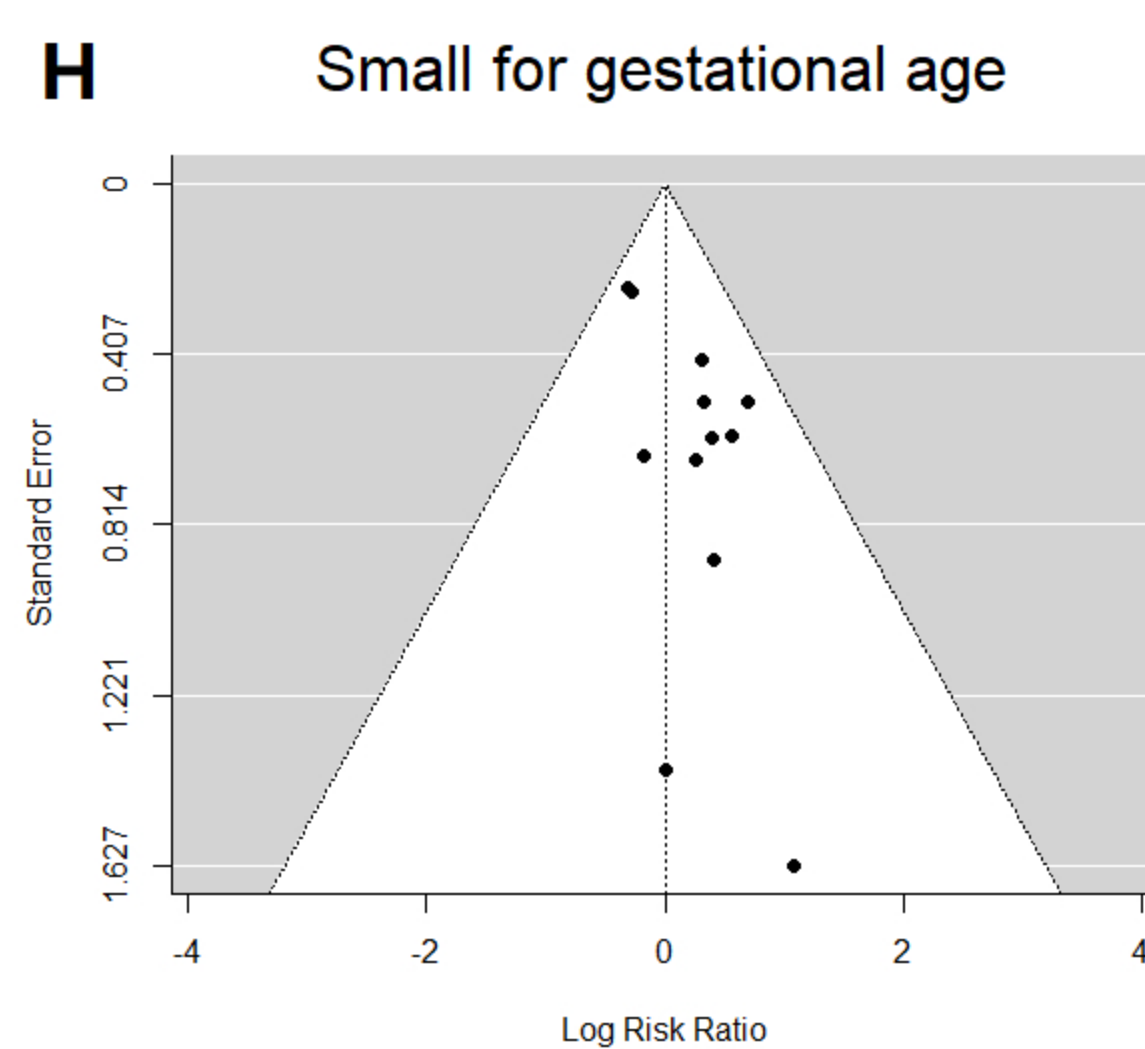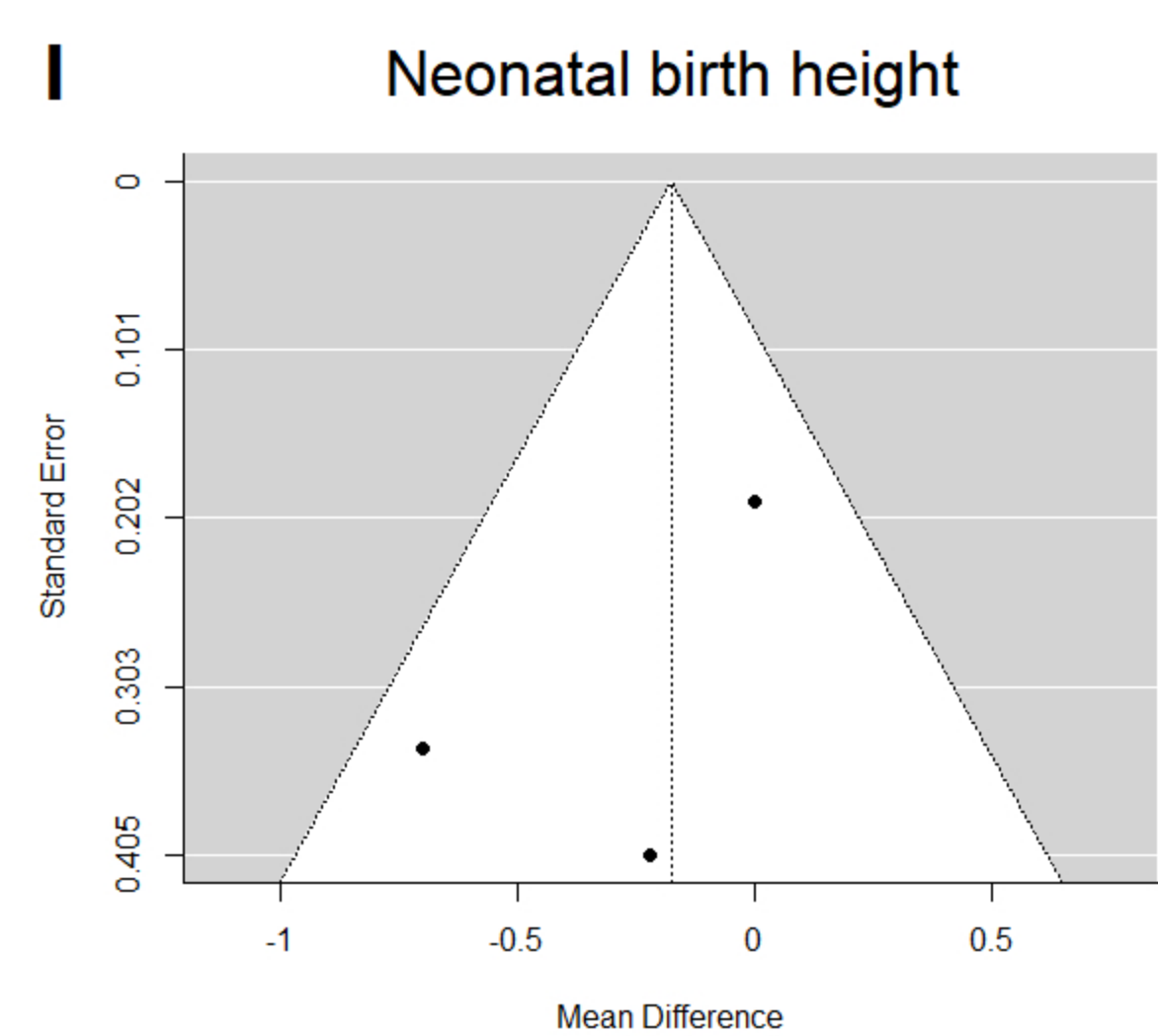

Supplement: Supplementary file 5 — Fig. S3. Funnel plots to assess publication bias of neonatal outcomes. (A) Neonatal birth weight. (B) Neonatal birth weight after trim-and-fill analysis. (C) Macrosomia. (D) Neonatal hypoglycemia. (E) Neonatal hypoglycemia after trim-and-fill analysis. (F) NICU admission. (G) LGA. (H) SGA. (I) Neonatal birth height. (PDF 873 kb) [file 592_2022_2016_MOESM5_ESM.pdf]

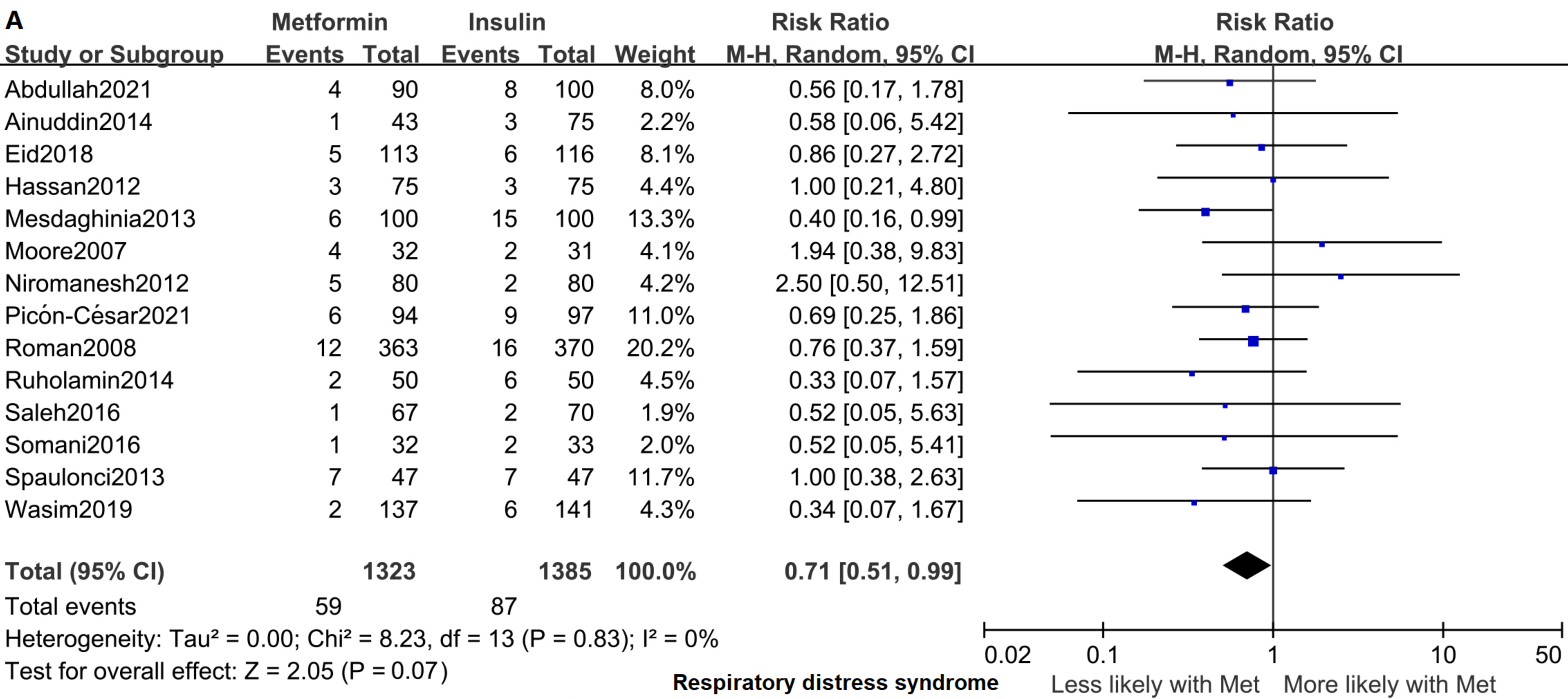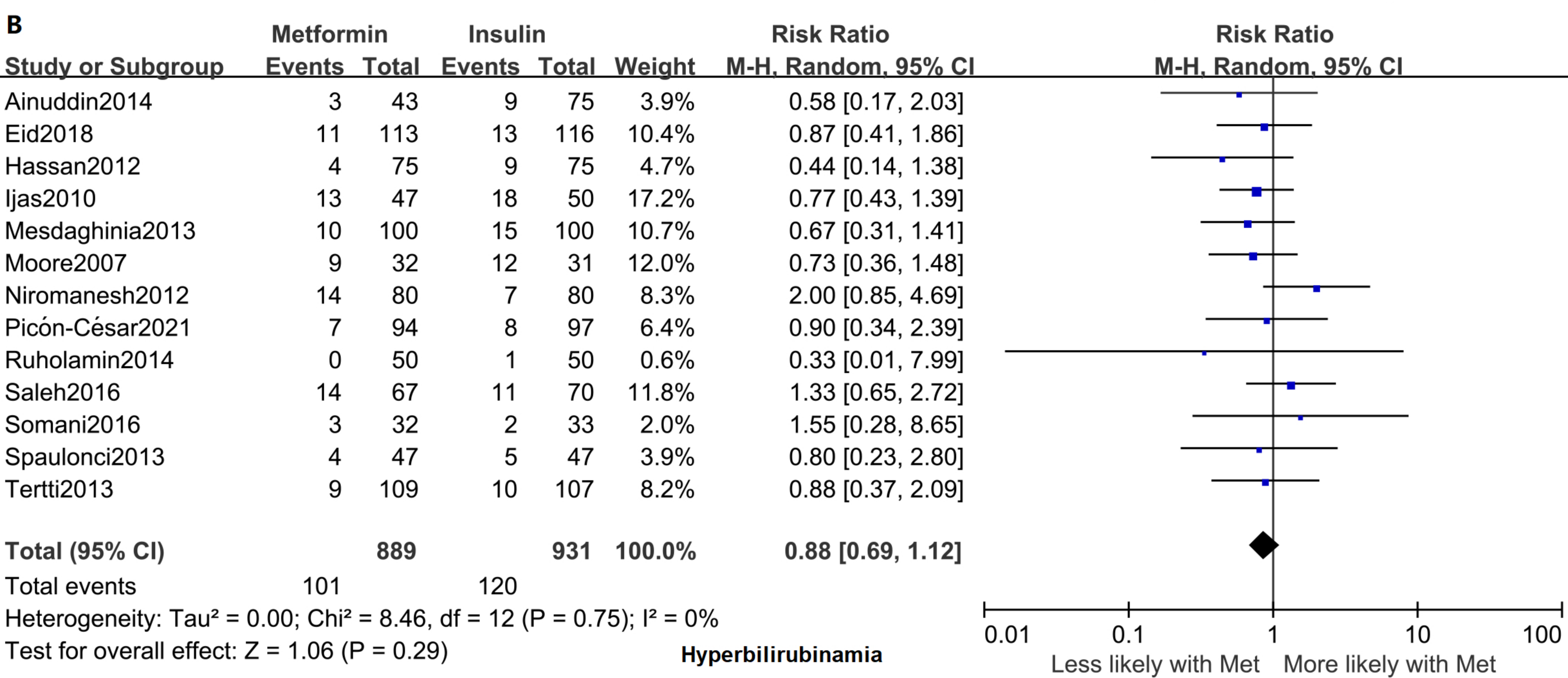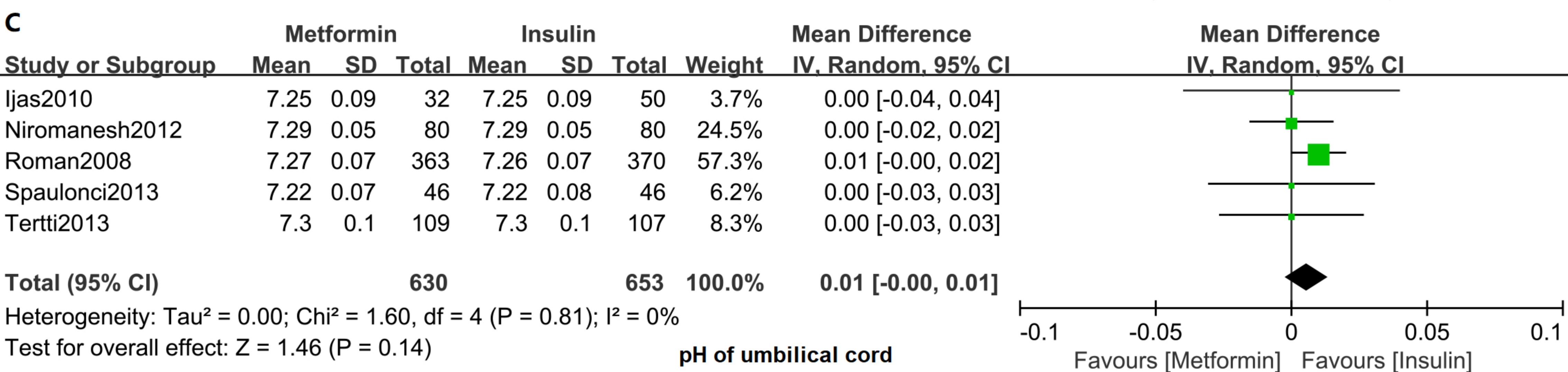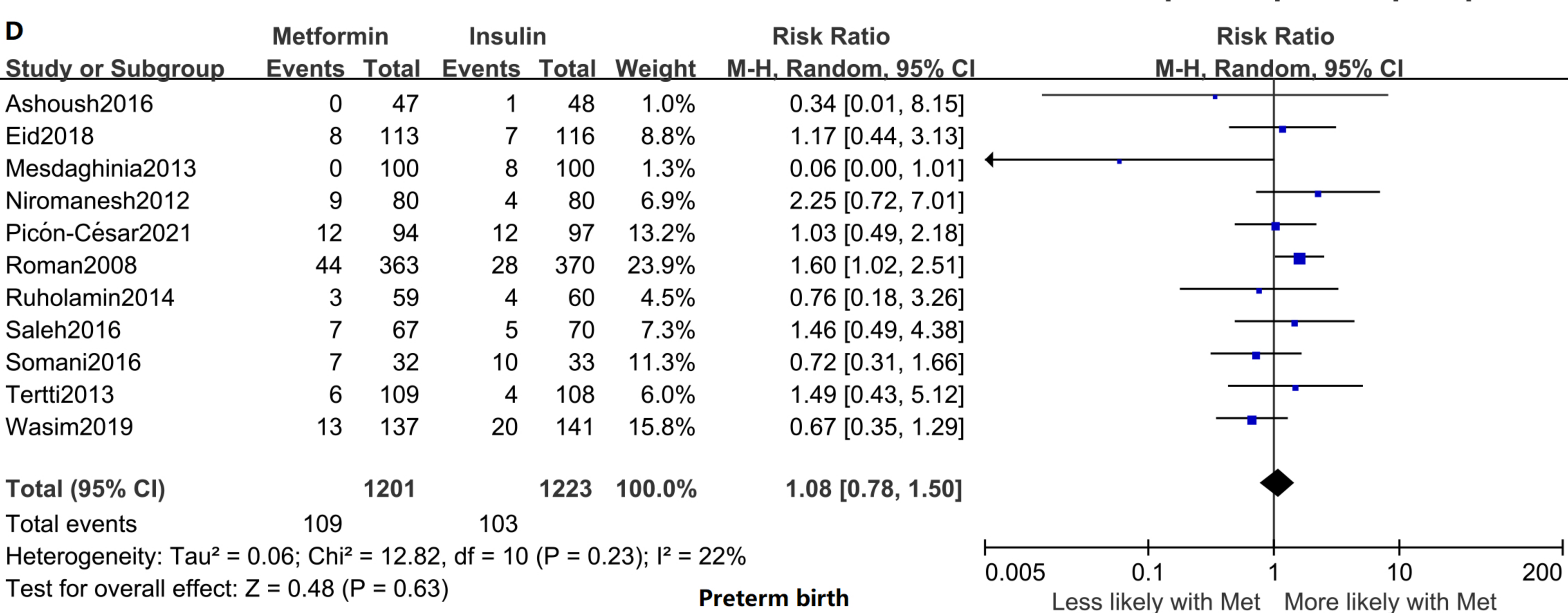

Supplement: Supplementary file 6 — Fig. S4. Forest plots for neonatal adverse outcomes. (A) Respiratory distress syndrome. (B) Hyperbilirubinemia. (C) Abnormal pH of umbilical cord. (F) Preterm birth. (PDF 2197 kb) [file 592_2022_2016_MOESM6_ESM.pdf]

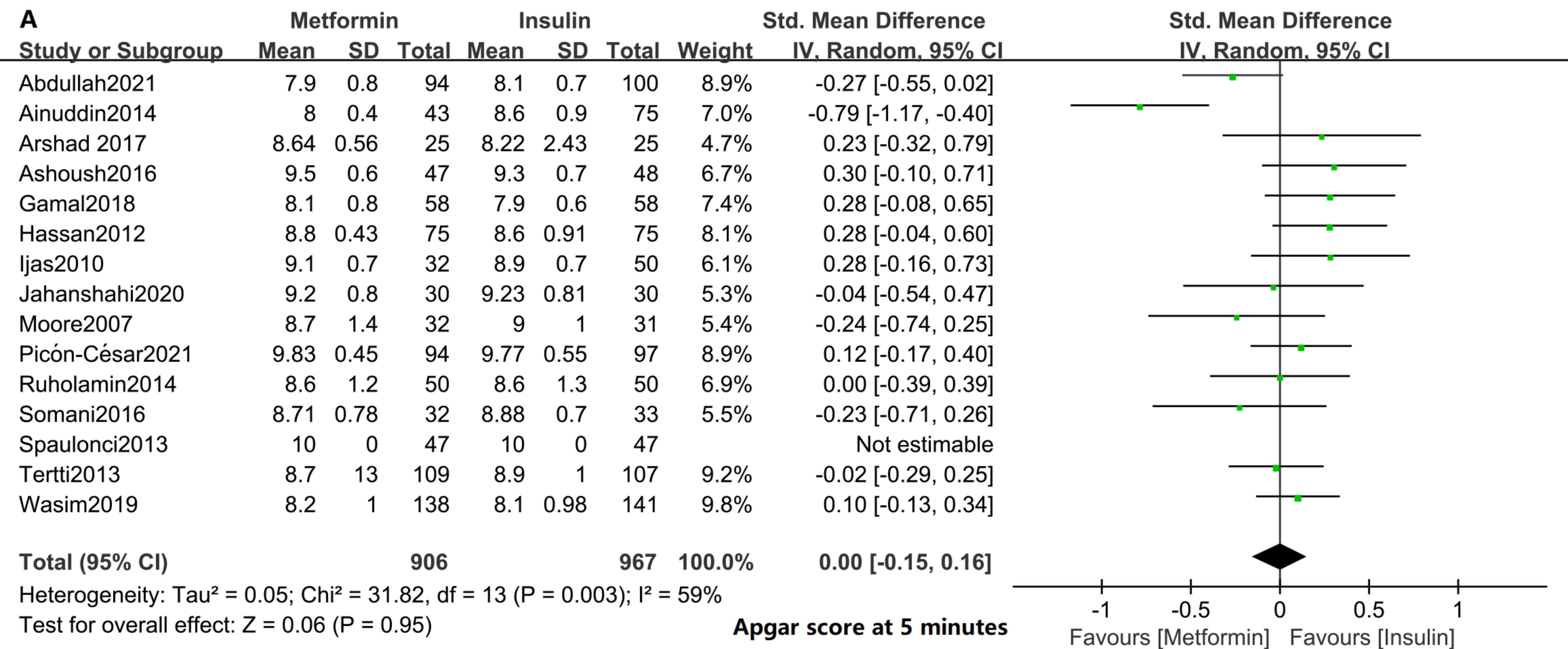

Supplement: Supplementary file 7 — Fig. S5. Forest plots for neonatal adverse outcomes. (A) Apgar score at 5 minutes. (B) Congenital anomalies. (C) Neonatal death. (D) Neonatal sepsis. (E) Birth trauma. (PDF 2421 kb) [file 592_2022_2016_MOESM7_ESM.pdf]
